# Halogen Atoms in the Protein–Ligand System. Structural and Thermodynamic Studies of the Binding of Bromobenzotriazoles by the Catalytic Subunit of Human Protein Kinase CK2

**DOI:** 10.1021/acs.jpcb.0c10264

**Published:** 2021-03-09

**Authors:** Honorata Czapinska, Maria Winiewska-Szajewska, Anna Szymaniec-Rutkowska, Anna Piasecka, Matthias Bochtler, Jarosław Poznański

**Affiliations:** † Institute of Biochemistry and Biophysics PAS, Pawińskiego 5a, 02-106 Warsaw, Poland; ‡ International Institute of Molecular and Cell Biology, Trojdena 4, 02-109 Warsaw, Poland; § Department of Biophysics, Institute of Experimental Physics, University of Warsaw, Pasteura 5, 02-089 Warsaw, Poland

## Abstract

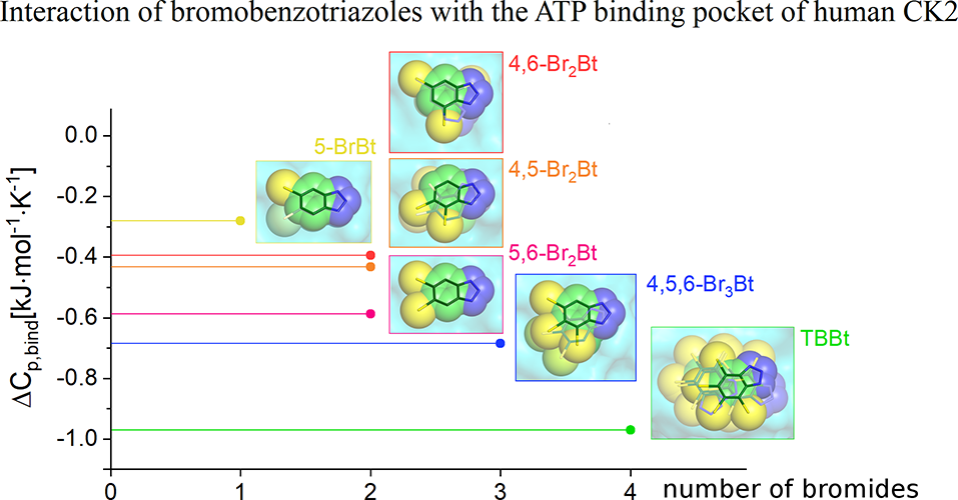

Binding of a family of brominated benzotriazoles to the catalytic subunit of human protein kinase CK2 (hCK2α) was used as a model system to assess the contribution of halogen bonding to protein–ligand interaction. CK2 is a constitutively active pleiotropic serine/threonine protein kinase that belongs to the CMGC group of eukaryotic protein kinases (EPKs). Due to the addiction of some cancer cells, CK2 is an attractive and well-characterized drug target. Halogenated benzotriazoles act as ATP-competitive inhibitors with unexpectedly good selectivity for CK2 over other EPKs. We have characterized the interaction of bromobenzotriazoles with hCK2α by X-ray crystallography, low-volume differential scanning fluorimetry, and isothermal titration calorimetry. Properties of free ligands in solution were additionally characterized by volumetric and RT-HPLC measurements. Thermodynamic data indicate that the affinity increases with bromo substitution, with greater contributions from 5- and 6-substituents than 4- and 7-substituents. Except for 4,7-disubstituted compounds, the bromobenzotriazoles adopt a canonical pose with the triazole close to lysine 68, which precludes halogen bonding. More highly substituted benzotriazoles adopt many additional noncanonical poses, presumably driven by a large hydrophobic contribution to binding. Some noncanonical ligand orientations allow the formation of halogen bonds with the hinge region. Consistent with a predominantly hydrophobic interaction, the isobaric heat capacity decreases upon ligand binding, the more so the higher the substitution.

## 1 Introduction

CK2, formerly designated as casein kinase II, is a pleiotropic serine/threonine kinase found in all eukaryotes.^1^ The holoenzyme is a heterotetramer, consisting of two catalytic α- and/or α′-subunits and two regulatory β-subunits.^2^ Unlike other kinases, in particular signaling cascade ones, CK2 is a constitutively active enzyme. It phosphorylates substrates with serine/threonine in regions enriched in acidic residues. The enzyme particularly favors targets with an acid or a phosphorylated serine residue at the +3 position relative to the substrate S/T.^1^ CK2 belongs to (or according to some classifications, is closely related to) CMGC kinases that in addition to CK2 also comprise cyclin-dependent kinases (CDKs), mitogen-activated protein kinase (MAPK), glycogen synthase kinase (GSK), and cyclin-dependent kinase-like kinases (CDK-like kinases).^3^

The physiological role of animal CK2 is understood at least partially from the loss of function phenotypes in mice.^3^ Disruption of the CK2 β-subunit gene leads to a cell-autonomous defect and early embryonic lethality, even though the noncatalytic β-subunits are not required for CK2 activity. The loss of either α- or α′-subunits leads to milder phenotypes, presumably due to the overlapping roles of the two variants. Among them, α seems to have a broader function than α′ since α is required for embryonic development,^4^ whereas α′ appears to be important only for spermatogenesis.^5^ Studies of mammalian CK2 substrates have identified a wide variety of proteins, including many targets involved in gene expression and protein synthesis but also signaling. CK2 appears to have a general antiapoptotic role.^6^ Thus, some cancer cells are more susceptible to CK2 inhibition than nonmalignant cells. Attempts to exploit this “CK2 addiction”^7^ are underway, using the orally available inhibitor Silmitasertib (CX-4945).^8−10^ Possible indications include gastric cancer,^11^ hematological malignancies,^12,13^ and cholangiocarcinoma.^14^

From a structural biology perspective, CK2 kinases are well characterized. The first structure was reported for a maize α-subunit^15^ and was followed by the structure of the human holoenzyme containing all four subunits.^16^ At present over 200 structures of CK2 enzymes with various inhibitors have been deposited in the public Protein Data Bank (PDB) repository, and many more are available in commercial databases. The CK2α subunit has a typical eukaryotic protein kinase (EPK) fold^17^ with a smaller N-terminal lobe consisting mostly of β-strands and the functionally important C-helix and a larger C-terminal lobe that is predominantly α-helical and harbors catalytically relevant loops. The active site region contains the typical elements for an EPK.^18,19^ Based on a large number of crystal structures and homology to PKA, a model for catalysis has been developed,^19^ involving two Mg^2+^ ions, Mg1^2+^ and Mg2^2+^, that coordinate the β- and γ- and α- and γ-phosphates of the ATP cosubstrate, respectively. Most catalytic residues are contributed from the C-terminal lobe. An aspartate (D175) from the DFG motif (DWG in hCK2α) coordinates Mg1^2+^ and Mg2^2+^. Another aspartate (D156), from the Y/HRD motif (HRD in hCK2α), is thought to accept a proton from the substrate during catalysis. An asparagine (N161) anchors Mg2^2+^. The N-terminal lobe of CK2α contributes a lysine (K68) from the N-loop and a glutamate (E81) from the C-helix. The lysine ε-amino group interacts with α- (and β-) phosphates of the ATP cosubstrate and is positioned by a salt bridge to the glutamate.

Several characteristic features distinguish CK2 from most other EPKs (as reviewed in refs (17 and 20)). As a constitutively active kinase, CK2 is typically, although with some exceptions,^21^ crystallized with the C-helix and activation loop in the active conformation.^20^ The active form is secured intramolecularly by an N-terminal segment that has been described as the functional equivalent of cyclins activating cyclin-dependent kinases.^20^ The hinge region between the N- and C-terminal lobes adopts a unique conformation that has been cited as the structural explanation for the CK2 specific promiscuity to accept either ATP or GTP as the cosubstrate for the phosphorylation reaction.^22^

Benzotriazoles and in particular their halogenated derivatives have long attracted interest because of their pharmacological properties. The best studied of these compounds is 4,5,6,7-tetrabromobenzotriazole (TBBt), the ATP-competitive inhibitor of type I (i.e., binding to the kinase in the active conformation).^23^ As the ATP pockets of EPKs in the active conformation are very similar,^18^ TBBt could be expected to inhibit a broad spectrum of kinases. Surprisingly, this is not the case. After the initial demonstration of its inhibitory activity against CK2 (and not CK1),^24^ it was soon shown to be highly selective for CK2, inhibiting to a significant extent only CDK2 and GSK3β of the CMGC group and PHK (phosphorylase kinase) of the CaMK family.^25^

Recently, much effort has gone into the design of bisubstrate analogue CK2 inhibitors that are based on benzotriazole scaffolds, often in the tetrabrominated form.^26−29^ Moreover, there have been efforts to extend the benzotriazole skeleton on the triazole side to better explore the full size of the purine-binding cavity.^30^ A derivative of tetrabromobenzimidazole (TBBi), SEL24-B849, which inhibits PIM and FLT3 kinases, is under development as a potential drug for hematological malignancies.^31^

The promising pharmacological profile of TBBt has triggered our extensive investigations of the entire series of halogenated and particularly brominated benzotriazoles.^32−37^ We have determined the inhibitory activity of all nine possible isomers of bromobenzotriazole against hCK2α,^32^ as well as their binding affinity to the enzyme (using several complementary biophysical methods).^33−35^ The studies demonstrated that 4,5,6- and 5,6-bromobenzotriazoles are nearly as potent as inhibitors of hCK2α as TBBt. In contrast, 5-, 4,5-, 4,6-, and 4,5,7-bromobenzotriazoles bind hCK2α with substantially lower affinities, and 4,7- and 4-bromobenzotriazoles virtually do not bind to the enzyme. We have also determined physicochemical parameters of the free ligands in aqueous solution, including solubility, p*K*_a_ for the dissociation of the triazole proton,^32^ and a large set of hydrophobicity descriptors, including the recently proposed solute excess volume.^36^

Structural information on the binding mode of halogenated benzotriazoles to CK2 is complex. Structures of TBBt bound to the α-subunit of maize CK2 (mCK2α)^38^ and human CDK2/cyclin A kinase^39^ have been reported. A structure of hCK2α in complex with 3-(4,5,6,7-Br_4_-1*H*-benzotriazol-1-yl)propan-1-ol, which can be considered a nonionizable TBBt analogue, has also been determined.^40^ The structures of mCK2α in complex with TBBi^41^ and pentabromoindazole^42^ that differ in the nitrogen configuration in the heterocyclic ring are known. Structures of benzotriazole compounds with other bromination patterns bound in the hCK2α pocket are to our knowledge not available. However, the structures of 5,6-bromobenzotriazole (5,6-Br_2_Bt) in complex with mCK2α^36^ and of the chloro analogue of 5,6-Br_2_Bt (5,6-Cl_2_-1-*b*-D-ribofuranosyl-benzimidazole, DRB) bound to hCK2α^43^ have been published.

Several hundred halogenated ligands have already been identified in publicly available protein kinase structures.^44^ Although they mainly bind to the ATP-binding site, their poses are significantly different.^39,41,44,45^ For halogenated benzotriazoles and benzimidazoles, prior studies have indicated that the binding mode is determined by a tug of war between a hydrogen bond/salt bridge and halogen bonding.^41^ The estimation of the free energy of a halogen bond in real biological systems is still controversial since the introduction of a halogen atom (Cl, Br, I) into a ligand molecule affects many of its physicochemical properties, including hydrophobicity, polarity, and p*K*_a_ of nearby ionizable groups. Our recent thermodynamic studies on binding of variously brominated benzotriazoles to hCK2α have supported the concept of the predominant contribution of electrostatic interactions and ligand (de)solvation to the free energy of binding.^33^

Here, we present a set of crystal structures of eight halogenated benzotriazoles in complex with a catalytic subunit of human protein kinase CK2. One of the nine studied ligands, 4,5,7-Br_3_Bt, was not reliably detected in the hCK2α ATP-binding pocket in the cocrystal. The structural analysis is accompanied by thermal denaturation and calorimetric data that show an increase of ligand affinity with bromo substitution. The thermodynamic data also indicate a decrease of isobaric heat capacity upon ligand binding, attributable to the hydrophobic contribution to complex formation.

## 2 Methods

### 2.1 Reagents

Brominated benzotriazoles were obtained as described previously.^33,46^ The catalytic domain of human protein kinase (hCK2α) was expressed and purified according to the published procedure.^33^ Protein sample homogeneity was confirmed by gel electrophoresis. The thermal profile of fluorescence-monitored protein stability was used prior to the calorimetric experiment as a descriptor of the proper folding of the enzyme.^34^

### 2.2 Crystallization

hCK2α was stored at −80 °C in glycerol solution prior to use. Before crystallization, the protein was dialyzed against buffer A (25 mM Tris-HCl pH 8.5, 0.5 M NaCl, 5 mM β-mercaptoethanol). The protein sample was then concentrated on VivaSpin 50000 MWCO to 4–8 mg/mL and mixed with a 0.25 M solution of a ligand in DMSO in 1:24 molar ratio. Crystals were grown in sitting drops by the vapor diffusion method. The 2 μL drops were set up manually using the 1:1 mixture of protein–ligand complex solution and crystallization buffer (0.1 M sodium HEPES/MOPS buffer pH 7.5, 20 mM sodium formate, 20 mM ammonium acetate, 20 mM sodium citrate tribasic dihydrate, 20 mM sodium potassium tartrate tetrahydrate, 20 mM sodium oxamate, 20% poly(ethylene glycol) 550 monomethyl ester, 10% poly(ethylene glycol) 20000). Crystals could be flash-cryocooled without an additional cryoprotection step. Diffraction data were collected at the P14 beamline of EMBL/DESY (Hamburg, Germany), P11 beamline of DESY (Hamburg, Germany), and MX 14.1 beamline of BESSY (Berlin, Germany). Preliminary diffraction experiments were also carried out at the XRD1 beamline of ELETTRA (Trieste, Italy).

### 2.3 Structure Determination

The structures were readily solved by molecular replacement with the help of the MolRep program^47^ using the structure of the human holoenzyme as a model (PDB code 3WAR(48)). Subsequently, they were rebuilt with the help of ARP/wARP^49^ and refined with the REFMAC program.^50^ In the cases of multiple not-easily resolvable ligand poses, the position of the ligands was confirmed using anomalous difference maps generated with the phases obtained from the Fourier transformation of the hCK2α structures with the ligands omitted. The ligand constraints were generated with the PRODRG server.^51^ Since some of the crystals showed signs of weakly broken symmetry and could only be integrated with a unit cell two times larger than that observed in the other data, the *R*_free_ reflections were selected in thin resolution shells. Data collection and refinement statistics are summarized in Table 1. The refined models and the corresponding structure factors were deposited in the PDB with the accession codes stated in the table.

**Table 1:** Data Collection and Refinement Statistics

data collection	hCK2α- 4-BrBt	hCK2α-5-BrBt	hCK2α-4,5-Br_2_Bt	hCK2α-4,6-Br_2_Bt	hCK2α-4,7-Br_2_Bt	hCK2α-5,6-Br_2_Bt	hCK2α-4,5,6-Br_3_Bt	hCK2α-TBBt (4,5,6,7-Br_4_Bt)
space group	*P*4_2_2_1_2	*P*4_2_2_1_2	*P*4_2_2_1_2	*P*4_2_2_1_2	*P*4_2_2_1_2	*P*4_2_2_1_2	*P*4_2_2_1_2	*P*4_2_2_1_2
cell dimensions								
*a*, *b* (Å)	128.5	128.8	129.3	128.0	128.3	127.2	129.0	127.4
*c* (Å)	61.1	60.9	60.7	61.1	61.2	60.9	61.1	61.0
wavelength (Å)	0.9794	0.9116	0.9794	0.9794	0.9116	0.9117	0.9116	0.9116
resolution (Å)	1.73	1.67	1.81	1.46	1.64	1.93	1.69	1.88
lowest shell	(44.3–5.15)	(36.5–4.98)	(36.5–5.38)	(44.2–4.36)	(44.3–4.89)	(36.2–5.72)	(44.4–5.03)	(45.0- 5.60)
highest shell	(1.83–1.73)	(1.77–1.67)	(1.92–1.81)	(1.55–1.46)	(1.74–1.64)	(2.04–1.93)	(1.79–1.69)	(1.99–1.88)
*R*_meas_ (%)^a^	5.6 (3.2, 193.2)	9.1 (5.0, 128.7)	7.8 (5.4, 104.4)	5.3 (3.9, 200.3)	6.6 (3.5, 191.0)	14.2 (3.8, 170.3)	10.2 (5.4, 166.1)	9.1 (5.5, 127.8)
CC1/2^a^	100 (99.9, 83.1)	99.9 (99.9, 85.6)	99.9 (99.8, 93.8)	100 (99.9, 72.6)	100(100, 77.9)	99.9 (100, 80.7)	99.9 (99.9, 83.1)	99.9 (99.9, 85.1)
*I*/σ*I*^a^	32.1 (103.5, 1.94)	20.6 (53.8, 2.11)	22.8 (55.6, 2.12)	32.1 (90.1, 1.99)	29.0 (85.31, 2.1)	22.7 (82.5, 1.98)	19.5 (52.3, 1.96)	20.6 (51.1, 2.07)
completeness (%)^a^	99.0 (99.6, 98.6)	99.2 (99.4, 95.5)	98.9 (99.3, 93.6)	99.9 (99.8, 99.6)	98.3 (97.4, 96.7)	99.9 (99.4, 99.5)	97.9 (99.6, 86.7)	99.6 (99.7, 97.6)
multiplicity^a^	24.6 (20.6, 20.7)	24.4 (22.2, 22.2)	24.7 (21.8, 23.7)	25.1 (21.9, 22.4)	26.2 (22.8, 26.4)	26.2 (23.3, 25.4)	25.1 (22.1, 24.3)	24.8 (21.3, 23.6)
no. reflections	53681	59111	46962	88427	62198	38226	57252	41198
refinement								
*R*_work_	16.02	14.44	16.39	15.39	14.86	15.33	14.88	15.97
*R*_free_	18.73	17.26	18.45	17.27	17.76	18.86	17.13	18.70
no. atoms^b^	3728	4076	3643	4152	3795	3855	3995	3799
protein	3244	3435	3196	3517	3233	3299	3405	3283
ligand	10	30	33	33	33	11	36	65
other	474	610	414	597	529	545	554	452
bond lengths rmsd (Å)	0.007	0.008	0.007	0.006	0.007	0.005	0.006	0.005
bond angles rmsd (deg)	1.40	1.44	1.39	133	1.36	1.27	1.35	1.25
Ramachandran								
allowed (%)	100	99.7	100	100	100	100	100	100
favored (%)	96.4	96.7	96.8	97.3	96.7	96.4	95.8	96.7
mol probity clash score	2.2	3.9	3.8	3.1	2.5	2.3	3.8	3.6
PDB code	6TLW	6TLV	6TLU	6TLS	6TLR	6TLP	6TLO	6TLL

a Lowest and highest shell in parentheses.

b Alternative conformations counted separately.

### 2.4 Surface Area Calculations

The solvent accessible surface changes were estimated with the Yasara Structure package (www.yasara.com). All low-mass molecules other than ligands placed at the hCK2α ATP-binding site were removed prior to the calculations. The polar surface included all nitrogen and oxygen atoms together with their associated hydrogens, while the nonpolar one covered all remaining atoms with the exception of bromines, which were treated separately. The change of solvent accessible surface upon complex formation (ΔASA) was calculated as the difference between the surface of the complex and the sum of the surfaces of the ligand and the free protein calculated for crystal structures. The ΔASA values were estimated separately for each alternative position of the ligand and/or enzyme conformation and then averaged using occupancies as weights with RMSE values assigned as their inaccuracies.

### 2.5 Low-Volume Differential Scanning Fluorimetry (nanoDSF)

The assay was carried out in 25 mM Tris-HCl (pH 7.5, 0.5 M NaCl) buffer with the protein and ligands concentration preserved constant at 2.5 and 25 μM, respectively. The samples were loaded into nanoDSF grade Standard Capillaries (NanoTemper Technologies) and analyzed using the Prometheus NT.48 nanoDSF device (NanoTemper Technologies). Thermal unfolding of the protein was monitored using a linear thermal ramp (1 °C·min^–1^; 20–80 °C) with an excitation power of 30%. All numerical models were globally fitted to the experimental data, assuming a two-state cooperative transition at *T*_m_, using the Marquardt algorithm^52^ implemented in the Origin 2019 package (OriginLab, Northampton, MA; www.originlab.com) according to the following equations.
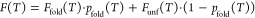

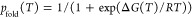

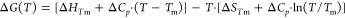

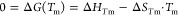
where *F*(*T*) is the observed fluorescence signal, *F*_fold_(*T*) and *F*_unf_(*T*) are the low- and high-temperature linear asymptotes of *F*(*T*); Δ*G*(*T*) is the free energy of unfolding at a given temperature; *T*_m_ is the middle-point transition temperature; Δ*H*_*T*m_ and Δ*S*_*T*m_ are the heat and entropy of the unfolding at *T*_m_ (Δ*G*(*T*_m_) = 0); and Δ*C*_*p*_ is the heat capacity change upon the protein unfolding.^53^

### 2.6 Isothermal Titration Calorimetry (ITC)

ITC measurements were carried out using the MicroCal iTC200 (Malvern) calorimeter. The hCK2α samples were diluted to the required protein concentration with the 25 mM Tris-HCl pH 8 buffer containing 0.5 M NaCl. Stock ligand solutions were diluted with the appropriate DMSO volume prior to mixing with the buffer to obtain the required ligand concentration and a final DMSO content of 1%. Due to the limited solubility of halogenated benzotriazoles, all titration experiments were performed in the reverse mode, in which buffered ligand solution was placed in the calorimetric cell and titrated with the protein solution.^35^ The resulting thermograms were preprocessed with the supplied MicroCal ITC-ORIGIN Analysis Software, and the thermodynamic parameters were further estimated at each temperature using previously reported customized procedures^54,55^ reimplemented in the Origin 2019 package (OriginLab, Northampton, MA; www.originlab.com).

For the three strongest ligands, the titration experiments were additionally performed using the NanoITC calorimeter (TA Instruments). We have found that heats of binding (Δ*H*) estimated using the two calorimeters differ significantly. To deal with these discrepancies, we have studied the reference reaction of 18-crown-6 with BaCl_2_ recommended by IUPAC (Table S1).

The dissociation constant was close to the reference value for both calorimeters, but the enthalpy for binding was consistent with the IUPAC value only for the MicroCal iTC_200_, while for the NanoITC it was underestimated by approximately 12%. Interestingly, a similar trend was reported by others for the titration of TRIS with HNO_3_.^56^ The inspection of the raw data, together with the technical specification and hardware-specific baseline determination algorithms, clearly suggests that the thermodynamic data from the MicroCal iTC_200_ are more reliable. Only these data were analyzed further, but for completeness we also report thermodynamic parameters determined with a NanoITC calorimeter (Table S2). In all experiments the maximum ligand concentration of 10 μM was used to minimize a possible contribution of the secondary binding site.

### 2.7 Density Measurements

Partial molar volumes were estimated with a density meter Anton Paar DMA 5000 M at 20, 25, and 30 °C from the concentration–density dependency for compounds dissolved in 50 mM phosphate buffer (pH 11). The experimental procedure and the data analysis method were described previously.^36,57,58^ The partial molar volume at 25 °C (*V*_2_^0^) and thermal volumetric expansivity of the solute (α_2_^0^ = ∂*V*_2_^0^/∂*T*) were estimated globally from two independent dilution series. The density of pure solvent (*d*_0_) was extrapolated individually for each experimental condition (i.e., temperature and buffer preparation). The change of *d*_0_ with temperature was further used to determine thermal volumetric expansivity coefficient of the bulk buffer according to the formula α_0_ = −(∂d_0_/∂*T*)/*d*_0_.

## 3 Results

### 3.1 Structural Analysis of hCK2α–Bromobenzotriazole Complexes

To verify the binding mode of brominated benzotriazoles to human CK2α, crystals of the enzyme in complex with eight differently halogenated ligands were obtained (covering all but one combination of benzyl ring modifications) (Table 1). Cocrystallization with the ninth compound (4,5,7-Br_3_Bt) was attempted, but the ligand was not detected in the ATP-binding pocket of the enzyme. The crystals belonged to the *P*4_2_2_1_2 tetragonal space group and contained a single molecule of the complex in the asymmetric unit. Probably due to the crystallization conditions, Mg^2+^ ions were absent from the active site of the kinase, with surprisingly little effect on the protein conformation including residues directly involved in the metal chelation. All complexes presented in this work show the hallmarks of the active CK2 form (Figure S1). In particular, the C-helix (residues 74–89) of the N-terminal lobe and the activation loop (residues 182–190) are precisely in the expected positions. However, the complex structures differ notably from the reference conformation of the active kinase by the disorder of the P-loop (glycine-rich loop, residues 45–49). In the presence of the cosubstrate ATP, the P-loop wraps over the triphosphate and is fairly ordered. In its absence, as in our structures, it lacks the anchor and becomes disordered (Figure S1).

**Figure 1:**
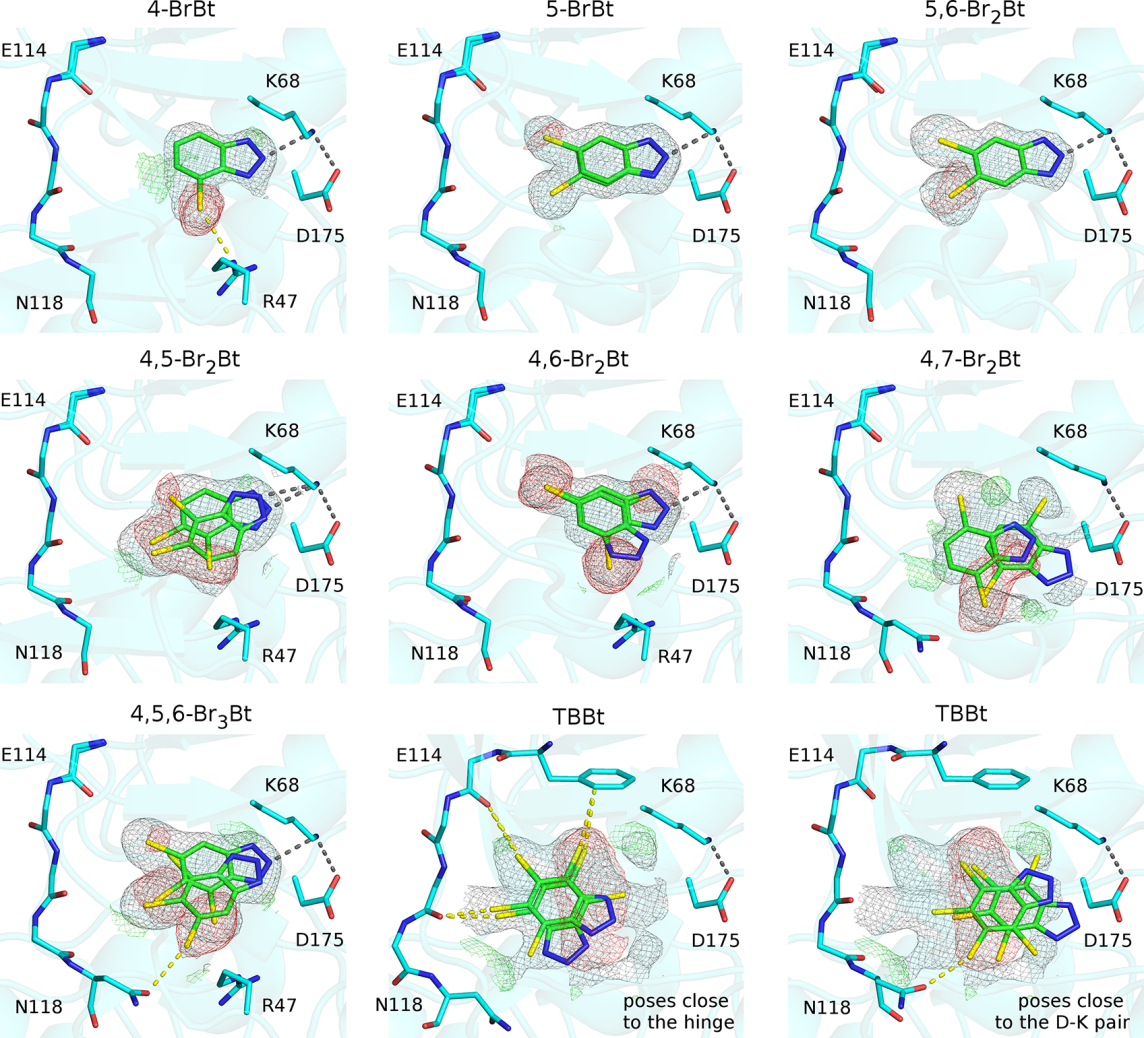
Ligand-binding pocket of human CK2α in complex with eight bromobenzotriazole inhibitors. Protein–ligand contacts including hydrogen bonds are indicated in gray and halogen interactions in yellow. The side chains of most hinge region residues and the main chains of R47, K68, and D175 are omitted for clarity. Composite omit (gray, contoured at 1 rmsd) and *F*_0_ – *F*_c_ (red/green contoured at ±3 rmsd) maps are shown for the ligand. The last two panels depict four poses observed for TBBt. With the exception of the compounds with 4,7-substitution, the bromobenzotriazoles can adopt a canonical pose. In many cases, particularly for the highly substituted benzotriazoles, multiple poses are observed.

All bound bromobenzotriazoles were located in the ATP-binding pocket (Figure 1). The ligand-binding modes were deduced from the composite omit maps and independently confirmed by the analysis of the crystallographic anomalous signal from the bromine atoms (Figure S3). In all eight structures, we have detected the signal from the bromine atoms even if the data were collected away from the absorption edge. In some cases, additional binding sites on the surface were observed, including a previously defined site located at the interface of α- and β-domains (Figure S2).^43^ Interestingly, a signature of a weak secondary binding site was also recently reported for several bromobenzotriazoles using a combination of MST and ITC.^35^

**Figure 2:**
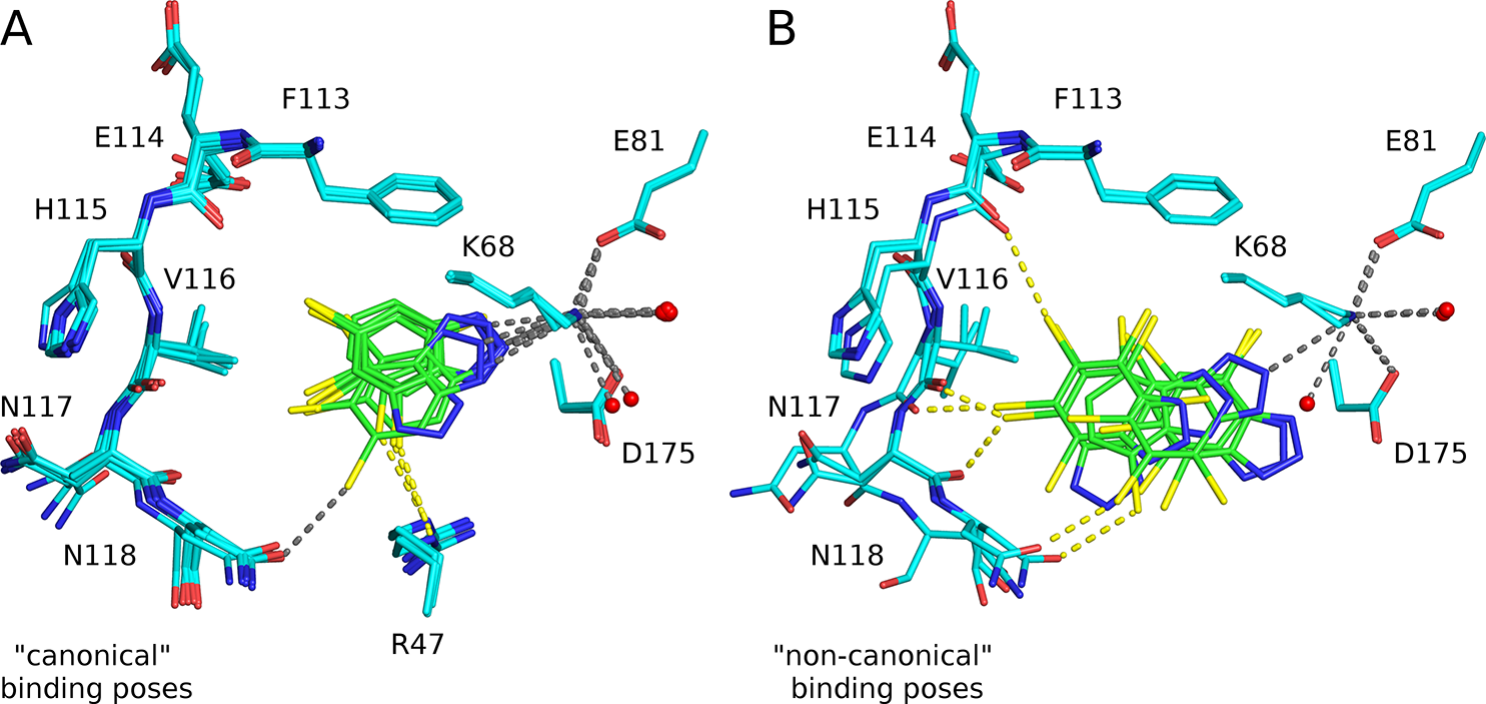
Binding poses of bromobenzotriazoles in the hCK2α ATP-binding pocket. For the canonical pose (A) hydrogen bonding or salt bridge formation is chosen over halogen bonding, while for some noncanonical poses (B) halogen-bonding interactions are formed, at the expense of the hydrogen bond or salt bridge.

The binding modes of bromobenzotriazoles in the hCK2α ATP-binding pocket are best described by dividing them into one “canonical” and several “noncanonical” ones. The canonical binding mode is observed for 4-BrBt, 5-BrBt, 4,5-Br_2_Bt, 4,6-Br_2_Bt, 5,6-Br_2_Bt, and 4,5,6-Br_3_Bt (Figure 2). The benzotriazoles in this group, except for 5-BrBt and 4,5-Br_2_Bt, bind in one predominant pose, similar for all of them. Additional binding modes are also observed for 4,6-Br_2_Bt and 4,5,6-Br_3_Bt, but they are clearly weaker than the dominant one. The binding mode of 5-BrBt in the crystal structure resembles that of 5,6-bromobenzotriazole. However, the ligand is observed in two alternative positions. The single 5-bromo substituent is located more or less deep in the ATP-binding pocket and corresponds to either the 5- or 6-bromo substituent of 5,6-Br_2_Bt. The identity of the 5-BrBt is unambiguously confirmed by the secondary binding site in which only one ligand pose is observed. For 4,5-Br_2_Bt, one of the binding modes is similar to the canonical one, and the second one is 180° rotated, so that the positions of the 5-bromo substituent and the N8 nitrogen are roughly preserved but the ligand is flipped.

The canonical binding pose of bromobenzotriazoles in the hCK2α-binding pocket orients the triazole toward the active site lysine (K68) and the phenyl ring toward the hinge region. Depending on the charge states of the lysine and the ligand in the pocket (highly brominated benzotriazoles are expected to be acidic), this binding pose creates the possibility for a hydrogen bond or a salt bridge with the side chain of K68. For the benzotriazoles with 5- and/or 6-bromo substituents, there is also a chance for the formation of one or two halogen bonds with carbonyl oxygen atoms of the kinase hinge region as the acceptors. Since the potential hydrogen (or salt bridge) and halogen-bonding partners are typically too distant to each other for both interaction types to occur simultaneously, a tug of war between them is set up. Not unexpectedly, this competition is resolved in favor of the hydrogen bond or salt bridge formation, judging from the distances in the crystal structures. In the cases where this is possible (for compounds with the 4-bromo substituent), the halogen bond may instead be formed with one of the conformers of R47 or N118. The arginine is anchored in the P-loop region that in these cases adopts at least two distinct conformations; the asparagine belongs to the hinge region.

The noncanonically binding bromobenzotriazole group is comprised of the compounds with both 4- and 7-bromo substituents present (4,7-Br_2_Bt and TBBt). Ligands from this group are pushed away from the pocket by the presence of the substituent in the “lateral” position. As a result, the hydrogen bond/salt bridge formation is more difficult, and the interaction is sometimes lost in favor of a halogen bond or optimized shape complementarity. The 4,7-Br_2_Bt ligand is bound in one dominant and one weaker mode. TBBt in turn, possibly due to its disclike shape, explores essentially the entire pocket, in multiple and not clearly separable poses. In some of the orientations, the benzotriazole is closer to the hinge region, so that halogen bonds may be formed. However, the lack of discrimination between different poses of this ligand in the CK2α pocket suggests that the hydrophobic effect is the dominant driver for its binding.

### 3.2 Stabilization of hCK2α by the Bromobenzotriazoles

**Figure 3:**
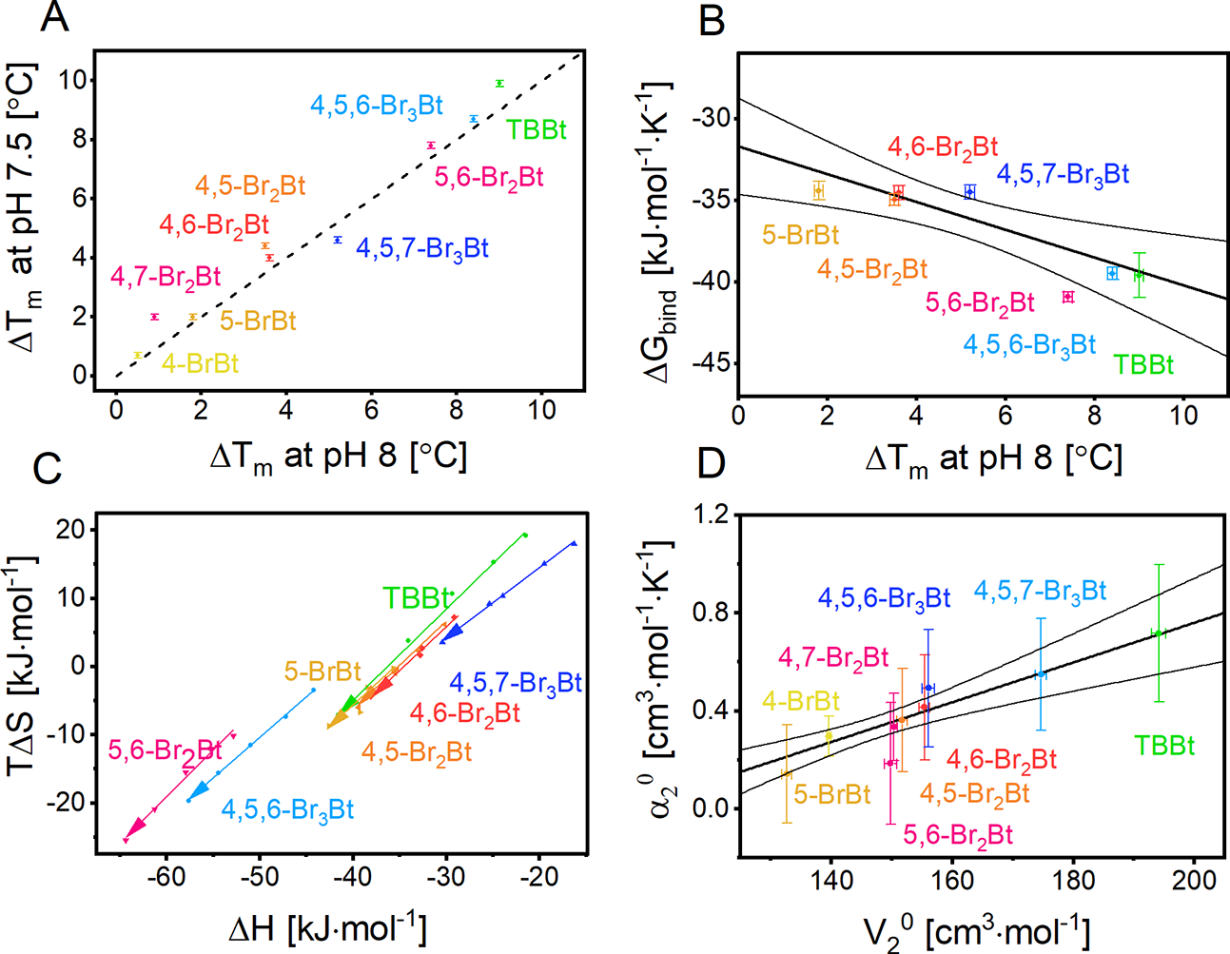
Thermodynamics of the hCK2α–bromobenzotriazole interaction. (A) Thermal unfolding of hCK2α complexes was monitored by nanoDSF at pH 7.5 and 8.0. Δ*T*_m_ values indicate the difference of the unfolding temperature in the presence and absence of a ligand. The dashed line shows the expectation for no pH dependence. The more highly substituted bromobenzotriazoles stabilize the protein more strongly, indicating tighter binding. (B) Correlation of the free energies of binding (at 25 °C) with hCK2α stabilization. Thin lines represent 95% confidence bands for the fitted line. (C) Entropy–enthalpy compensation with respect to temperature. The arrows mark the general trend in the temperature range from 15 to 35 °C. Entropic Δ*S* and enthalpic −Δ*H* contributions decrease and increase strongly with temperature, respectively. The two effects mostly compensate for the free energy of binding Δ*G* = Δ*H* – *T*Δ*S*. Figures S6 and S7 show detailed trends observed for the particular ligands. (D) Correlation of the solute thermal expansivity with the partial molar volume of ligands.

Recent comprehensive thermodynamic^33^ and biochemical studies^32^ allowed us to rank bromobenzotriazole ligands in terms of their binding affinity to hCK2α. The results clearly demonstrated that a balance of hydrophobic and electrostatic interactions has predominant contribution to the binding affinity.^32−34^ The structures of hCK2α complexes shown above as well as the published values of IC_50_ (Table 2)^32^ were determined at pH 7.5, while the previous thermodynamic studies were performed at pH 8.0.^33,35^ This prompted us to measure the thermal stabilities of hCK2α and its complexes at pH 7.5 by fluorescence-monitored thermal denaturation (nanoDSF) (Table 2). The extent of ligand-induced hCK2α stabilization was almost the same at both pH values (Figure 3A). It grew with the number of bromo substituents, with larger contributions from the modifications in 5 and 6 than 4 and 7 positions. We have previously shown that the dissociation constants determined for halogenated benzotriazoles, when converted according to the ATP concentration in the assay, perfectly reconstruct experimental IC_50_ values.^33^ Similarly, the ITC-derived binding affinities generally agreed with the inhibitory activities determined for the same ligands^32^ (Figure S4).

**Table 2:** Thermal Stability of hCK2α in Complex with Nine Bromobenzotriazole Compounds Determined with Fluorescence-Monitored Thermal Denaturation (nanoDSF) at pH 7.5 and 8.0^33^ and Literature Data for the Inhibitory Activity of the Studied Ligands^32^

ligand	*T*_m_ (°C) at pH 7.5	Δ*T*_m_ (°C) at pH 7.5	Δ*T*_m_ (°C) at pH 8^33^	IC_50_ (μM) at pH 7.5^32^
apo	45.19 ± 0.03	–	–	–
4-BrBt	45.92 ± 0.05	0.7 ± 0.1	0.5 ± 0.1	119 ± 10
5-BrBt	47.17 ± 0.02	2.0 ± 0.1	1.8 ± 0.1	26 ± 3
4,5-Br2Bt	49.55 ± 0.05	4.4 ± 0.1	3.5 ± 0.1	10.6 ± 0.9
4,6-Br_2_Bt	49.21 ± 0.04	4.0 ± 0.1	3.6 ± 0.1	10.0 ± 2.2
4,7-Br_2_Bt	47.15 ± 0.03	2.0 ± 0.1	0.9 ± 0.1	72 ± 11
5,6-Br_2_Bt	52.96 ± 0.05	7.8 ± 0.1	7.4 ± 0.1	0.56 ± 0.02
4,5,6-Br_3_Bt	53.84 ± 0.04	8.7 ± 0.1	8.4 ± 0.1	0.38 ± 0.02
4,5,7-Br_3_Bt	49.81 ± 0.07	4.6 ± 0.1	5.2 ± 0.1	5.8 ± 0.9
4,5,6,7-Br_4_Bt (TBBt)	55.11 ± 0.03	9.9 ± 0.1	9.0 ± 0.1	0.27 ± 0.07

### 3.3 Calorimetric Analysis of the Bromobenzotriazole–hCK2α Interaction

Thermodynamic parameters of the benzotriazole hCK2α complex formation were measured on the MicroCal iTC_200_ (Malvern). All ligands could be analyzed, with the exception of two weakly binding ones (4,7-Br_2_Bt and 4-BrBt). As expected, the calorimetric data confirmed the benzotriazoles with bromo substituents in the 5 and 6 positions (TBBt, 4,5,6-Br_3_Bt and 5,6-Br_2_Bt) as the strongest ligands. Tighter-binding ligands (more negative Δ*G* values) stabilized hCK2α more strongly (Figure 3B). The binding of the three strongest ligands was additionally followed with a NanoITC calorimeter (Table S2, Figures S5 and S6).

We observed the usual entropy–enthalpy compensation. For all ligands, the free energies of binding (Δ*G*) were only weakly temperature-dependent, despite the strong temperature dependence of enthalpies (Δ*H*) and entropies (Δ*S*). At higher temperatures, the enthalpic contribution for ligand binding increased, while the entropic change became more unfavorable (Figure 3C, Figures S6 and S7). The slope values of the *T*Δ*S*(Δ*H*) linear trend varied in the narrow range of (0.99 ± 0.03), clearly identifying that these two contributions compensate for the free energy of binding (Δ*G* = Δ*H* – *T*Δ*S*).

Entropy–enthalpy compensation was also observed for the number of bromo substituents in the ligand series. At fixed temperature, the binding enthalpy was less favorable for the more substituted brominated compounds. However, this effect was more than compensated by the increase in the entropic contribution. The strong entropic component can be interpreted as a signature of the hydrophobic effect: binding of the ligand releases more water molecules involved in the ligand solvation (and present in the ligand-binding cavity) into bulk solvent and thus increases the conformational freedom of these water molecules.

Since the isobaric heat capacity of water solvating the hydrophobic surfaces is greater than for bulk water (despite the misleading description of this phenomenon as the “iceberg” effect^59,60^), the binding of hydrophobic ligands should be accompanied by a decrease in the isobaric heat capacity (Δ*C*_*p*,bind_ < 0). Indeed, binding of brominated benzotriazoles was accompanied by negative Δ*C*_*p*,bind_ values (Table 3). The effect was more pronounced for highly substituted benzotriazole ligands than for less substituted ones. However, the Δ*C*_*p*,bind_ values did not follow the binding affinity. Instead, the ligands could be clustered into three groups, each with a different number of bromine atoms attached to the 5 and 6 position of the benzotriazole (Figure S8). All strong hCK2α ligands (*K*_d_ ∼ 0.1 μM; Δ*G*_bind_ ∼ −40 kJ·mol^–1^) were substituted at both C5 and C6; their weakly binding analogues (*K*_d_ ∼ 1 μM; Δ*G*_bind_ ∼ −35 kJ·mol^–1^) carried a single bromine at C5/C6, while those unsubstituted at C5/C6 virtually did not bind hCK2α.

**Table 3:** Free Energy of Binding at 25 °C (Δ*G*_bind_) and Heat Capacity Changes (Δ*C*_*p*,bind_) Associated with Bromobenzotriazole Interaction with hCK2α Determined at pH 8 with the Aid of Isothermal Titration Calorimetry (ITC)

ligand	Δ*G*_bind_ (kJ·mol^–1^)	Δ*C*_*p*,bind_ (kJ·mol^–1^·K^–1^)	ΔASA (Å^2^)	log *P*^36^	α_2_^0^ (cm^3^·mol^–1^·K^–1^)	*V*_2_^0^ (cm^3^·mol^–1^)	log(τ)^37^
4-BrBt			370 ± 2	1.72	0.30 ± 0.08	139.6 ± 0.3	–0.65
5-BrBt	–34.4 ± 0.6	–0.28 ± 0.13	376 ± 5	1.73	0.14 ± 0.20	132.7 ± 0.8	–0.41
4,5-Br_2_Bt	–34.9 ± 0.4	–0.43 ± 0.05	404 ± 3	2.34	0.36 ± 0.21	151.7 ± 0.9	–0.39
4,6-Br_2_Bt	–34.5 ± 0.4	–0.44 ± 0.06	391 ± 6	2.37	0.41 ± 0.21	155.4 ± 0.9	–0.38
4,7-Br_2_Bt			419 ± 1	2.37	0.19 ± 0.25	149.8 ± 1.0	–0.55
5,6-Br_2_Bt	–40.9 ± 0.3	–0.59 ± 0.07	395 ± 1	2.36	0.33 ± 0.14	150.0 ± 0.6	–0.30
4,5,6-Br_3_Bt	–39.5 ± 0.4	–0.68 ± 0.06	428 ± 4	2.96	0.49 ± 0.24	156.0 ± 1.0	–0.20
4,5,7-Br_3_Bt	–34.5 ± 0.4	–0.68 ± 0.07		2.98	0.55 ± 0.23	174.7 ± 0.9	–0.22
4,5,6,7-Br_4_Bt	–39.6 ± 1.4	–0.97 ± 0.10	479 ± 24	3.58	0.72 ± 0.28	194.1 ± 1.1	–0.00
Pearson’s *r* with Δ*C*_*p*,bind_			0.933	0.941	0.930	–	0.983
			(0.980)^a^	(0.994)^a^	0.949^*b*1^/0.980^*b*2^	0.999^*b*1^/0.997^*b*2^	

a Pearson’s *r* with Δ*C*_*p*,bind_ with values for ligands with fractional dissociation <0.95 (5-BrBt and 5,6-Br_2_Bt) excluded.

b Ligands were divided into two groups: ^*b*1^ the ones with both C5 and C6 positions brominated and ^*b*2^ the remaining ones.

### 3.4 Partial Molar Volume and Apparent Thermal Expansivity of Bromobenzotriazoles in Solution

According to the Lum, Chandler, and Weeks (LCW) theory, the hydrophobic interactions scale for solutes of subnanometer size with the volume of the molecule rather than its surface.^61^ We have recently proposed partial molar volume as the extensive parameter to describe both the solute volume and the solute-induced reorganization of the solvation shell.^36,57,58,62,63^ By analyzing density data at three temperatures (20, 25, and 30 °C), we could estimate the partial molar volume of the ligand (*V*_2_^0^) and the bulk solvent density (*d*_0_(*T*)). We could further separate the thermal expansivity coefficient of the bulk solvent (α_0_) and the apparent expansivity of the solute (α_2_^0^). The latter parameter is comprised mainly of the thermal expansion effect of the hydration shell. The values of α_0_ fell within the narrow range of (26.9 ± 1.3) × 10^–5^ K^–1^, which is close to the literature data for water (25.72 × 10^–5^ K^–1^ at 25 °C).^64^ The density estimated at 25 °C for independent buffer preparations varied minutely in the range of 1.00399–1.00403 g·cm^–3^, significantly exceeding the water density at 25 °C, 0.9970449 g·cm^–3^.^64^ The volumetric data concerning solute (*V*_2_^0^, α_2_^0^) are listed in Table 3. The solute thermal expansivity (α_2_^0^), together with the change of isobaric heat capacity upon ligand binding *(*Δ*C*_*p*,bind_), has already been associated with the properties of the solvation layer.^65,66^ Importantly, α_2_^0^ is proportional to the partial molar volume *V*_2_^0^ (Figure 3D). So, *V*_2_^0^ may be another descriptor of hydrophobic interactions. Consequently, the volume-normalized solute expansivity coefficient (α = α_2_^0^/*V*_2_^0^) is almost the same for all studied bromobenzotriazoles (232 ± 22 × 10^–5^ K^–1^). Importantly, this value substantially exceeds the thermal expansivity coefficient of the bulk buffer (α_0_ = 26.9 ± 1.3 × 10^–5^ K^–1^), clearly confirming that water organization changes with temperature more near the solute surface than in the bulk.

## 4 Discussion

### 4.1 Comparison of the Canonical Bromobenzotriazole hCK2α Complexes with Prior Structures

Benzotriazole and purine scaffolds are almost isostructural. Compared to the purines in ATP or GTP,^67−69^ the canonically bound benzotriazoles are located deeper in the pocket and further away from the hinge region (Figure S9AB). For benzotriazoles with bromo substituents in the 5 or 6 positions, the displacement avoids a steric conflict with the hinge (V116). Instead, one of the 5 or 6 halogen atoms adopts a position similar to that of the N6 atom of adenine. In the case of adenine, the exocyclic N6 amino group donates a hydrogen bond to the carbonyl oxygen atom of E114. For 5- or 6-substituted bromobenzotriazoles the σ-hole of the bromine atom could mimic electron-accepting properties of the N6 amino group. However, in the canonical hCK2α bromobenzotriazole complex structures, the distances between the backbone carbonyl oxygen atoms of E114 (and V116) from the hinge region of hCK2α and the bromine atoms are longer than the 3.4 Å sum of van der Waals radii of oxygen and bromine.^70^ Therefore, their location in the crystal structure is not indicative of halogen bonding. Instead the triazole part of the ligands tends to occupy a position close to that of the ATP α-phosphate oxygen atom and thus mimic its ionic interaction with the K68 at the bottom of the pocket.

### 4.2 “Noncanonical” Bromobenzotriazole hCK2α Complexes vs Prior Structures

TBBt and 4,7-Br_2_Bt are bound less deeply in the pocket of hCK2α compared to the canonical pose because the ligand would otherwise clash with the pocket walls. As a result, at least the tetrabromobenzotriazole tends to overlap better with the inferred binding of the ATP purine (Figure S9CD). The ligand not only binds in a shallower manner but also comes closer to the hinge, at least in some of the binding poses. This opens up the possibility of halogen bonding with the main chain carbonyl oxygen atoms of the hinge region. In this respect, the noncanonical binding mode of the benzotriazoles is similar to the binding mode of tetrabromobenzotriazole analogues with extra steric bulk on the triazole side, which are also pushed closer to the hinge region.^30^ It is instructive to compare the binding modes of TBBt to human CK2α with previously observed ones. All previous studies of TBBt complexes with maize CK2α^38^ and human CDK2^39^ and of a TBBt derivative with human CK2α′^45^ have reported a single ligand -binding pose. Interestingly, structural alignment shows that the alternative poses observed for TBBt in this work share the same space, with particularly good overlap of the halogen atom locations. The orientation of TBBt and its analogues in almost all complexes of CK2 with bisubstrate inhibitors^26−29^ is also consistent with the poses observed in our structure (Figure S10). The multiple TBBt poses observed by us and others suggest that the precise binding mode is not well-defined for this ligand. Thus, the thermodynamic contribution associated with the burial of its large hydrophobic surface in the nonpolar ATP-binding pocket of the kinase may predominate over specific interactions. This conclusion is in line with the review demonstrating that hydrophobic contacts determine the binding affinity (inhibitory activity) of halogenated benzotriazoles and benzimidazoles to protein kinases.^41^ Moreover, the “noncanonical” locations of TBBt display short contacts between bromine atoms and carbonyl oxygen atoms of E114 and V116 (Figures 1 and 2, poses close to the hinge). This is in line with our recent thermodynamic study of the four possible isomeric forms of dibromo–dichlorobenzotriazole, of which 5,6-Br_2_-4,7-Cl_2_Bt has the highest affinity to hCK2α.^37^ This result suggests that, at least in solution, the binding of perhalogenated benzotriazoles might be accompanied by two halogen bonds involving substituents in positions 5 and 6.

### 4.3 Hydrophobic Effect and the Decrease in the Isobaric Heat Capacity

**Figure 4:**
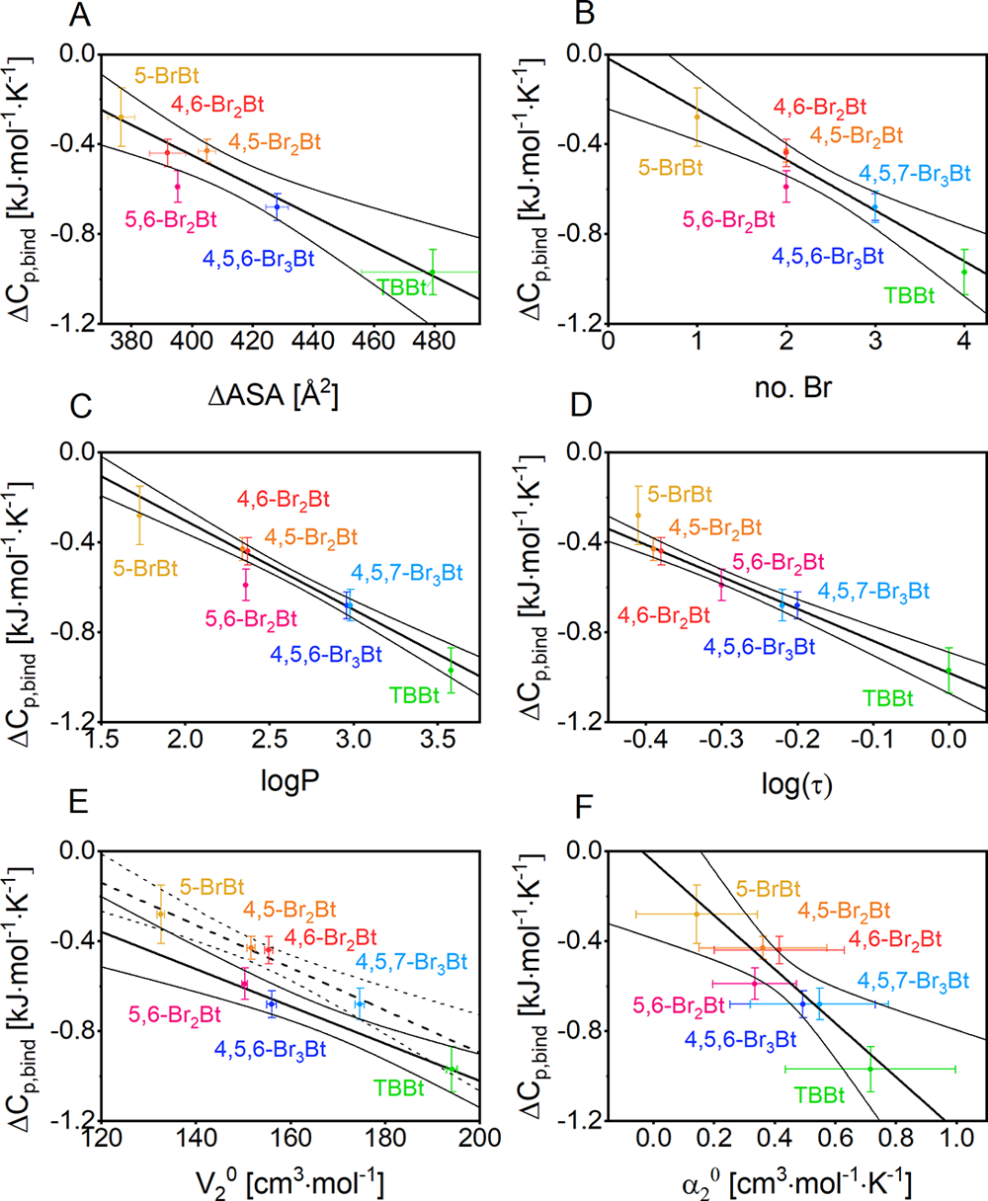
Heat capacity changes (Δ*C*_*p*,bind_) associated with the hCK2α–bromobenzotriazole interaction. Δ*C*_*p*,bind_ values were correlated with (A) the change in the solvent accessible surface of the ligand and the protein, (B) number of bromo substituents, (C) calculated water–octanol partition coefficient log(*P*),^36^ (D) previously measured HPLC-derived hydrophobicity data, log(τ),^37^ and (E, F) *V*_0_^2^ and α, respectively. In (C) and (E) 5,6-Br_2_Bt was excluded from the regression analysis. Note that the scale on the abscissa does not start at 0. None of the relationships is therefore a proportion, but the dependence of Δ*C*_*p*,bind_ on the number of bromo substituents comes close to such a relationship. Thin lines represent 95% confidence bands for the fitted line.

The affinity of bromobenzotriazoles to hCK2α increases with the number of bromine atoms. Halogen substitutions in the 5 and 6 positions have larger effects than those in the 4 and 7 positions. Thermodynamic data point to the hydrophobic effect as an important contributor to binding. The strong entropic component can be attributed to the release of highly ordered water molecules from the surfaces of the ligand and binding cavity. The most widely accepted signature of hydrophobically driven interaction is a decrease in the isobaric heat capacity (Δ*C*_*p*,bind_ < 0).^71^ Such a decrease is indeed observed in our experiments. In macromolecular crystallography, it is generally believed that for a large macromolecular surface the Δ*C*_*p*,bind_ values are proportional to the solvent accessible surface area (ΔASA) that is buried in the binding event. We have tested several parametrizations that correlate the reduction of polar and nonpolar solvent accessible surfaces upon ligand binding (ΔASA) with the heat capacity change associated with this process,^72−75^ but none of them properly predicted experimental values (Table S3, Figure S11A–D). Instead, we see a more pronounced drop in the heat capacity for the more highly substituted and therefore larger bromobenzotriazoles. The Δ*C*_*p*,bind_ value increases more strongly with the number of bromo substituents than a surface (or volume) argument suggests (Figure 4A). In the case of the number of bromo substituents as the independent variable, the correlation can be approximated by a linear relationship with no significant offset from the origin (Figure 4B). There are several explanations of this effect that are not mutually exclusive.

First, the Δ*C*_*p*,bind_ correction may depend on the degree of hydrophobicity. This factor may be similar to other measures of hydrophobicity, such as the water–octanol partition coefficient (log *P*)^36,37^ or the partial molar volume,^36^ even though molecular explanations for the Δ*C*_*p*,bind_ effect^76,77^ treat hydrophobicity as a qualitative rather than a quantitative property. Indeed, Spolar parametrization extended for bromine (3.47 J·mol^–1^·K^–1^·Å^–2^) reliably predicts all experimental Δ*C*_*p*,bind_ values (Figure S11E). This clearly indicates that hydrophobic contribution of Br is approximately 2.5-fold larger than that estimated for hydrocarbons (1.34 J·mol^–1^·K^–1^·Å^–2^).^73^ Second, electrostatic interactions with solvating water molecules decrease the heat capacity of the charged compounds.^78^ Upon binding events this will be reflected in heat capacity changes less negative than for the neutral counterparts. We have tested the dependence of Δ*C*_*p*,bind_ on the water octanol partition coefficient, log *P* (Figure 4C), and a hydrophobicity measure based on HPLC retention times, log(τ) (Figure 4D). Both dependencies can be described by linear regression. Two ligands (5-BrBt and 5,6-Br_2_Bt) were not consistent with the regression line relating Δ*C*_*p*,bind_ values and the commonly used *in silico* derived hydrophobicity indicators (ΔASA, log *P*). Since a relevant fraction of these two ligands is neutral at pH 8.0 (0.26 and 0.08, respectively),^46^ one may assume that the contribution of particular electrostatic interactions should be reflected in Δ*C*_*p*,bind_ values. Finally, the Δ*C*_*p*,bind_ change could also be due to the changes in protein conformation.

### 4.4 Entropic Balance and Conformational Change in the Protein upon Ligand Binding

Following the idea of Spolar and Record,^79^ the entropic change associated with ligand binding can be broken down into three main entropic components, the loss of rotational and translational degrees of freedom Δ*S*_rt_, the hydrophobic effect Δ*S*_HE_, and the reminder Δ*S*_other_. Due to the rigidity of the brominated benzotriazoles, Δ*S*_other_ is most likely attributable to changes in the protein conformation and order. The other two contributions to entropic change can be estimated with known formulas.

The entropy decrease resulting from the loss of rotational and translational degrees of freedom can be calculated based on the following equation.^80^ Please note that we have neglected a minute correction (∼6 J·mol^–1^·K^–1^) that allows for the difference between the temperature *T* of interest and the reference temperature *T*_ref_ for which the formula was originally developed (*m* is the molecular mass of the ligand):
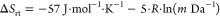


The change in heat capacity Δ*C*_*p*,bind_ predicts a proportional entropy change that depends on the logarithm of temperature.
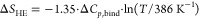


From the experimental dependence of the total entropy change on temperature, we can extrapolate the temperature *T*_s_ where the total entropy change is 0 (Figure S6DF and Table S4). At this temperature
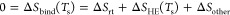
The Δ*S*_other_ contribution can be directly calculated using this expression (Table S4). Our data suggest that the Δ*S*_other_ contribution is relatively minor for TBBt, 4,5,6-Br_3_Bt, and 5,6-Br_2_Bt but equals 100–200 J·mol^–1^·K^–1^ for the other bromobenzotriazoles. According to Spolar and Record, the Δ*S*_other_ entropy change of ∼100 J·mol^–1^·K^–1^ is indicative for partial unfolding of 5–6 residues.^79^ Residues ^30^VVEWGNQD^37^ of hCK2α are disordered in the complexes with large Δ*S*_other_, but not in those with Δ*S*_other_ close to zero (Figure S12). This suggests that this region may be important for the protein contribution to entropic change, but since the structures include an almost 60 amino acid long disordered region at the C-terminus of the kinase (not visible in the crystal structures), this assignment remains speculative.

### 4.5 Grouping Brominated Benzotriazoles Based on Thermodynamic Data

According to the heat capacity upon ligand binding (Δ*C*_*p*,bind_) and partial molar volume (*V*_2_^0^), the bromobenzotriazoles cluster into groups that differ in the substitution pattern (Figure 4E, according to the F-test, the alternative model with no grouping must be rejected at *p* ∼ 10^–4^; *F*(1,5) = 57). Such a tendency may be discerned also for solute thermal expansivity (Figure 4F, according to the F-test, the alternative model with no grouping must be rejected at *p* = 0.023; *F*(1,4) = 12.6). Taken together, the thermodynamic data clearly show interplay between the direct hydrophobic effect, roughly represented by the number of bromine atoms in the molecule, and the halogen-induced changes of the ligand electronic properties related to the apparent polarity of the compound.

**Figure 5:**
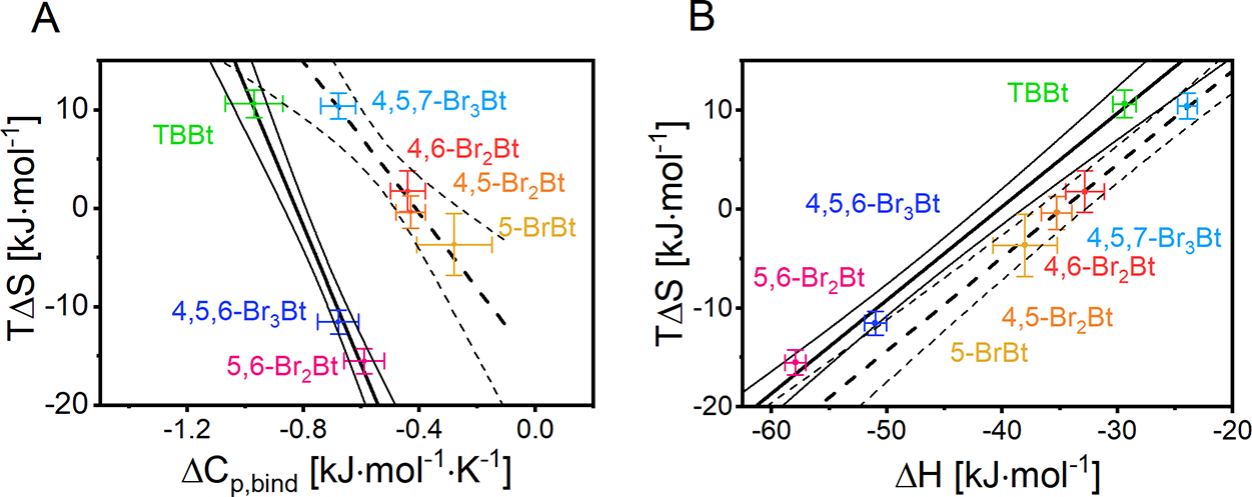
Correlation between principal thermodynamic parameters associated with ligand binding. (A) Two commonly accepted descriptors of the hydrophobic contribution to the free energy of ligand-binding cluster benzotriazoles into two groups. (B) The same groups are identifiable in entropy–enthalpy compensation observed at 25 °C. The two parallel lines are separated by ∼4.9 kJ·mol^–1^, which strictly corresponds to the free energy difference between strongly and weakly binding bromobenzotriazoles.

*T*Δ*S* and Δ*C*_*p*,bind_ are commonly used thermodynamic descriptors of the hydrophobic contribution to ligand binding. They cluster bromobenzotriazoles in the same groups (Figure 5A, according to the F-test, the alternative model with no grouping must be rejected at *p* ∼ 10^–4^; *F*(2,3) = 600). Similar trends have been reported for the IC_50_ values (Table 2).^32^ Moreover, the same regularity is observed for entropy–enthalpy compensation (Figure 5B, the alternative model assuming one group of ligands must be rejected at *p* = 0.003; *F*(1,4) = 43.7), for which two parallel lines of the 0.965 ± 0.026 slope indicate an almost perfect compensation among the grouped ligands. Such compensation is commonly observed for protein–ligand^81^ and protein–protein^82^ systems. The distance between the lines (estimated to be 4.9 kJ·mol^–1^ from the intercept values) reflects the difference in the free energy of protein–ligand interaction observed for strongly (5,6-Br_2_Bt, 4,5,6-Br_3_Br, TBBt) and weakly (5-BrBt, 4,5-Br_2_Bt, 4,6-Br_2_Bt, 4,5,7-Br_3_Br) binding ligands (Table 3). The systematic discrepancies strongly support the presence of two competing mechanisms of bromobenzotriazole hCK2α binding—one for high-affinity ligands, in which both 5 and 6 positions are brominated, and the other one for ligands in which one of the two positions remains unsubstituted.

## 5 Conclusions

Ligand-binding poses of brominated benzotriazoles in the CK2 active site are dictated by three competing drivers. These are salt bridge or hydrogen bond formation with K68, halogen bond formation with the linker region, and the hydrophobic effect. Salt bridge or hydrogen bond formation on the one hand and halogen bonding on the other hand are largely mutually exclusive because the hCK2α linker region and K68 are too far apart for both interactions to occur simultaneously. Salt bridge formation typically wins over halogen bonding unless the former is sterically disfavored (as in 4,7-substituted benzotriazoles). The hydrophobic effect plays a dominant role in bromobenzotriazole hCK2α binding. Its contribution is evidenced by the strong entropic drive (caused by the release of water molecules from the ligand surface into the bulk solvent) and by the negative Δ*C*_*p*,bind_. For the most highly brominated ligand (TBBt), the hydrophobic effect is so dominant that the ligand explores almost the entire cavity, thus forgoing in some poses the favorable interaction with K68.

## Supporting Information Available

Supplementary figures and tables providing more detailed depiction of the presented data (PDF)

## Accession Codes

Protein Data Bank (PDB) accession numbers: hCK2α-4-BrBt 6TLW, hCK2α-5-BrBt 6TLV, hCK2α-4,5-Br_2_Bt 6TLU, hCK2α-4,6-Br_2_Bt 6TLS, hCK2α-4,7-Br_2_Bt 6TLR, hCK2α-5,6-Br_2_Bt 6TLP, hCK2α-4,5,6-Br_3_Bt 6TLO, hCK2α-TBBt 6TLL.
