# Supplementary material for: Halogen Atoms in the Protein–Ligand System. Structural and Thermodynamic Studies of the Binding of Bromobenzotriazoles by the Catalytic Subunit of Human Protein Kinase CK2
Source: J Phys Chem B. 2021 Mar 9;125(10):2491–503. doi: 10.1021/acs.jpcb.0c10264 (PMC8041304; doi:10.1021/acs.jpcb.0c10264)
Supplement: Supplementary file 1 — jp0c10264_si_001.pdf [file jp0c10264_si_001.pdf]

Supplementary material for:

Halogen Atoms in The Protein-Ligand System. Structural and Thermodynamic Studies of The Binding of Bromobenzotriazoles by the Catalytic Subunit of Human Protein Kinase CK2.

Honorata Czapinska[1,2,$], Maria Winiewska-Szajewska[1,3,$], Anna Szymaniec-Rutkowska[1]

Anna Piasecka[1,2], Matthias Bochtler[1,2,*], Jarosław Poznański[1,*]

[1] Institute of Biochemistry and Biophysics PAS, Pawinskiego 5a, 02-106 Warsaw, Poland
[2] International Institute of Molecular and Cell Biology, Trojdena 4, 02-109 Warsaw, Poland
[3] Department of Biophysics, Institute of Experimental Physics, University of Warsaw, Pasteura 5, 02-089 Warsaw, Poland

[$] Equal contribution.
*Correspondence to be addressed to:
e-mail: jarek@ibb.waw.pl, tel: +48 22 5925783, fax: +48 22 5922190
e-mail: mbochtler@iimcb.gov.pl, tel: +48 22 5970732, fax: +48 22 5970715

Supplementary Figures

Figure S1.

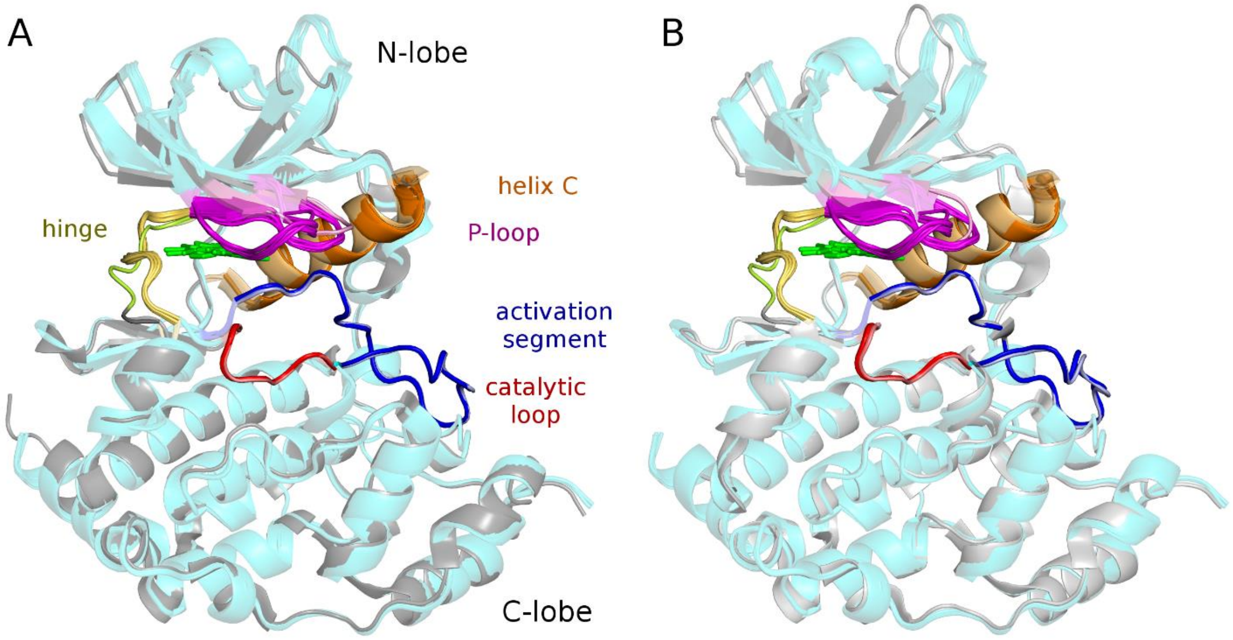

Figure S1. The superposition of the hCK2α bromobenzotriazole complexes determined in this work with (A) the enzyme in the absence of a co-substrate (dark gray, PDB code: 3AT2[1]) and (B) the enzyme in the presence of an ATP-analogue (light gray, PDB code: 3NSZ[2]). In the hCK2α inhibitor complexes, the hinge region is colored in yellow, the C-helix in orange, the P-loop in magenta, the activation segment in blue and the catalytic loop in red. The colors are fainter for the reference structures. The inhibitors are in green and the ATP analogue in faint green. Note the two conformations of the P-loop.

Figure S2.

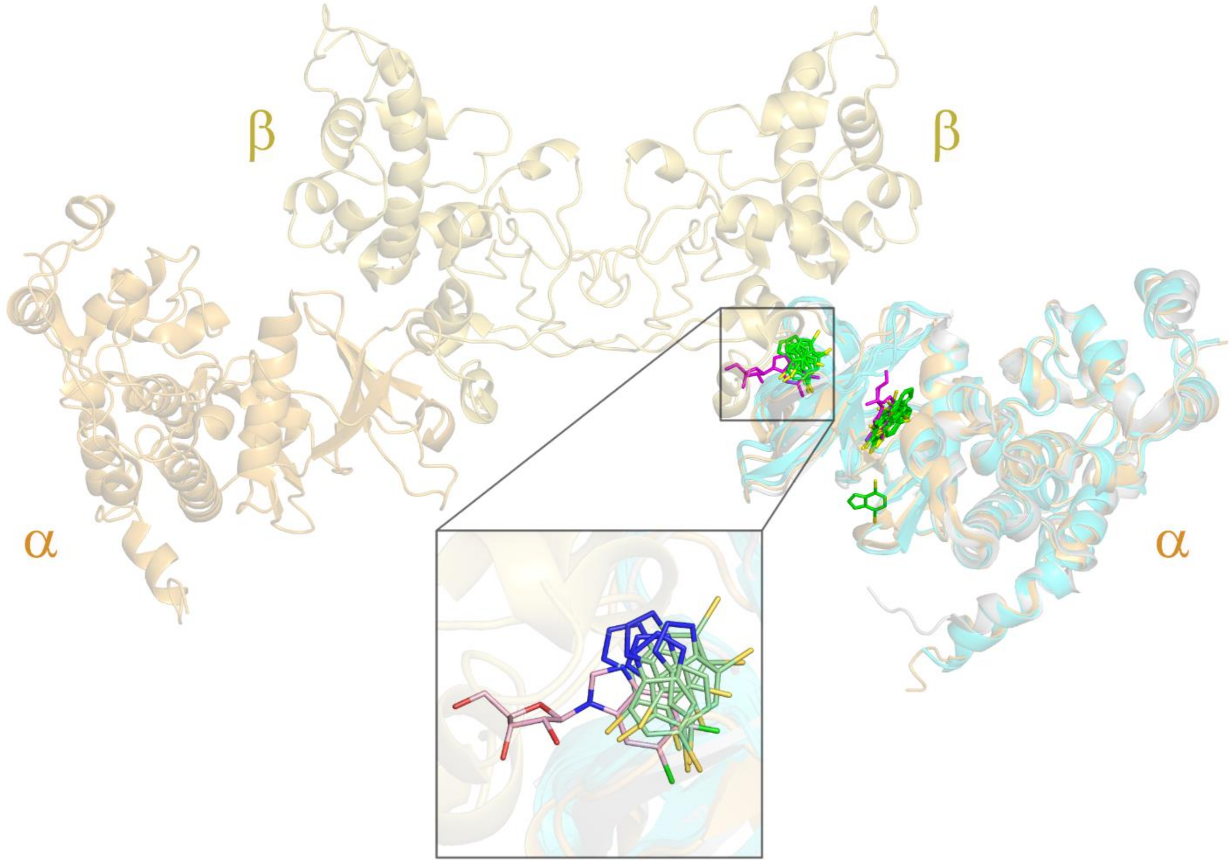

Figure S2. Alternative binding sites observed in the complexes of hCK2α with bromobenzotriazoles in comparison with previous structures. A single binding pose was modelled in an alternative binding site in the complexes of hCK2α with 4,5,6,7-Br_4Bt, 4,5,6-Br_3Bt, 4,6-Br_2Bt, 4,5-Br_2Bt and 5-BrBt. A different additional binding site was observed in the complex with 4,7-Br_2Bt. The structures presented here were overlaid with the complex of hCK2α with 5,6-Cl_2-1-b-D-ribofuranosylbenzimidazole (PDB code: 3H30[3]), and of the human CK2 holoenzyme (PDB code: 1JWH[4]).

Figure S3.

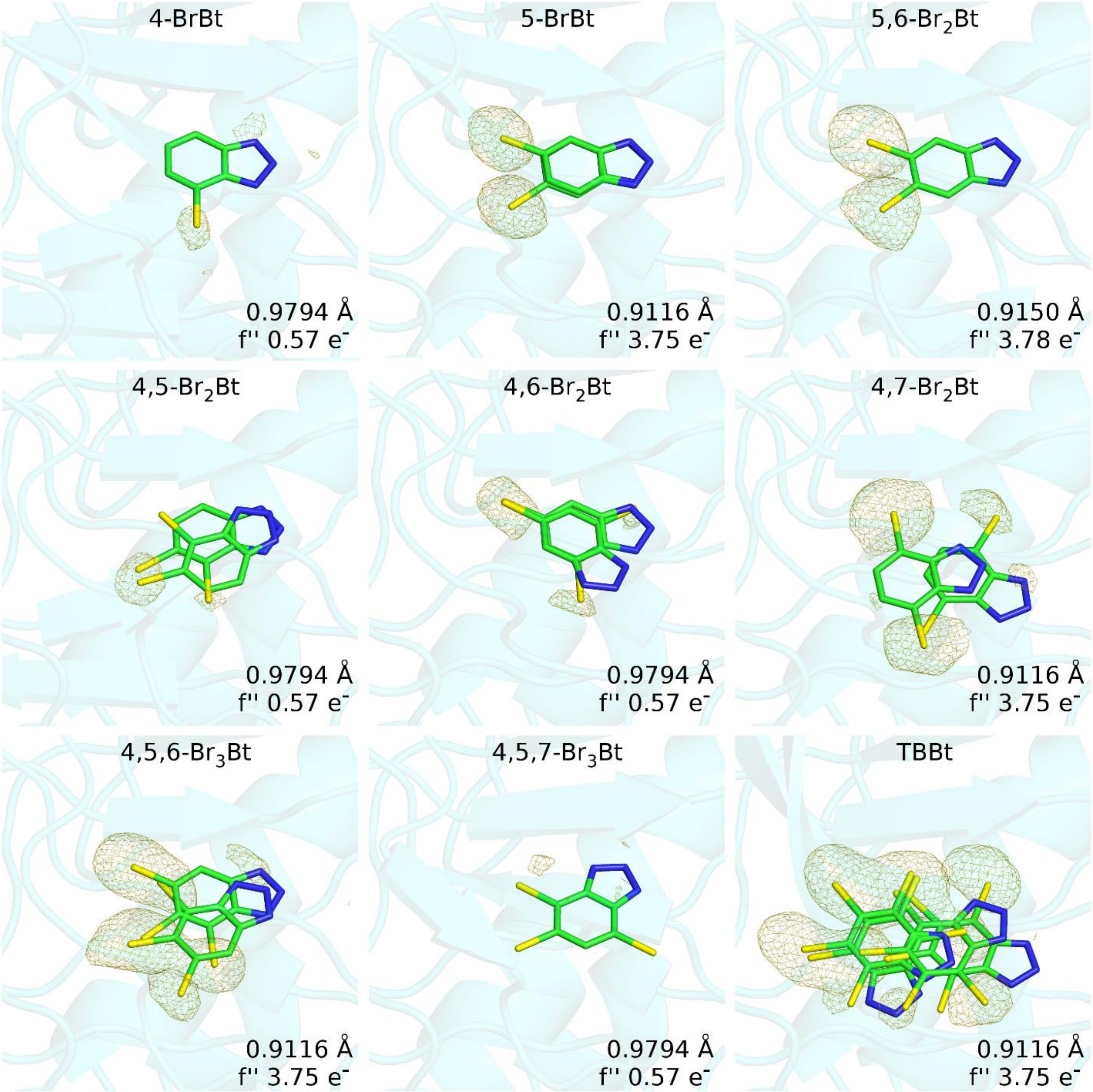

Figure S3. The anomalous signal confirming the bromide atom positions in the complexes of hCK2α with eight bromobenzotriazole inhibitors. The anomalous maps were contoured at 3 rmsd. Data collection wavelengths and the predicted strengths of anomalous signal are indicated. For the crystal of hCK2α obtained in the presence of 4,5,7-Br_3Bt, the anomalous signal could not be clearly detected. The ligand could not be reliably modelled based on the composite omit map and was likely not bound in the active site (a hypothetical ligand position is indicated).

Figure S4.

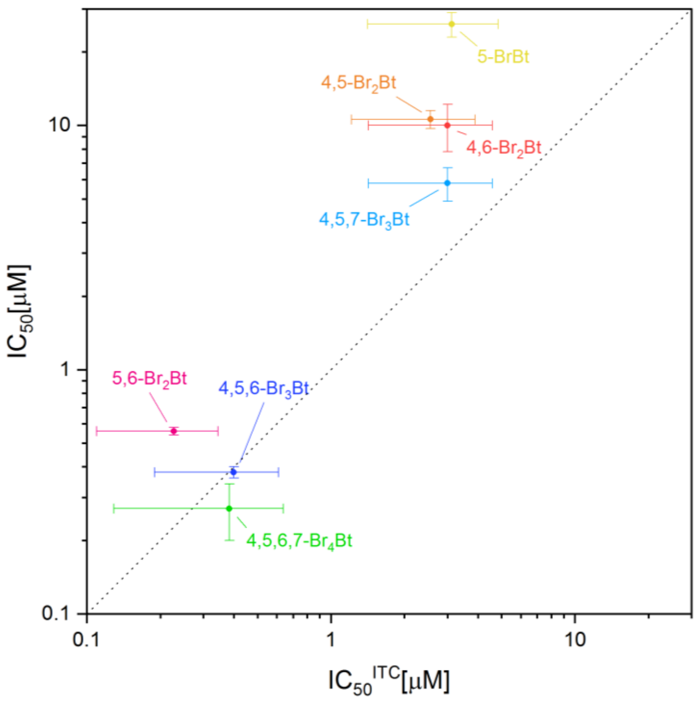

Figure S4. hCK2α inhibitory activity of halogenated benzotriazoles compared with the values predicted from their binding affinity determined with ITC. Assuming the competitive mechanism of hCK2α inhibition by benzotriazole derivatives, their inhibitory activity (IC_{50}) can be estimated directly from the binding affinity (K_d), according to the Cheng-Prusoff equation[5]

$$\mathrm{IC}_{50}^{ITC} = K_{\mathrm{d}} \cdot \left(1 + {[\mathrm{ATP}]}/{K_{\mathrm{ATP}}}\right)$$

where [ATP] is the ATP concentration in the assay and K_{ATP} is the dissociation constant of ATP. [ATP] = 10 μM and K_{ATP} = 4.3 ± 1.8 μM.[6] The disagreement between the predicted and measured IC_{50} values observed for 5-BrBt and 5,6-Br_2Bt must be assigned to the pH difference in both experiments (pH = 8.0 and 7.5, respectively), which affects the protonation equilibrium of these two ligands. This interpretation is further supported by MST data determined at pH 7.5,[6] for which such deviation has not been observed. For weakly binding ligands, calorimetrically derived IC_{50} values are systematically lower than IC_{50} values derived from inhibition experiments. The effect is likely due to the presence of a second binding site (Fig. S2). Binding to this site is expected to contribute to the ITC signal, but not to the inhibition.

Figure S5.

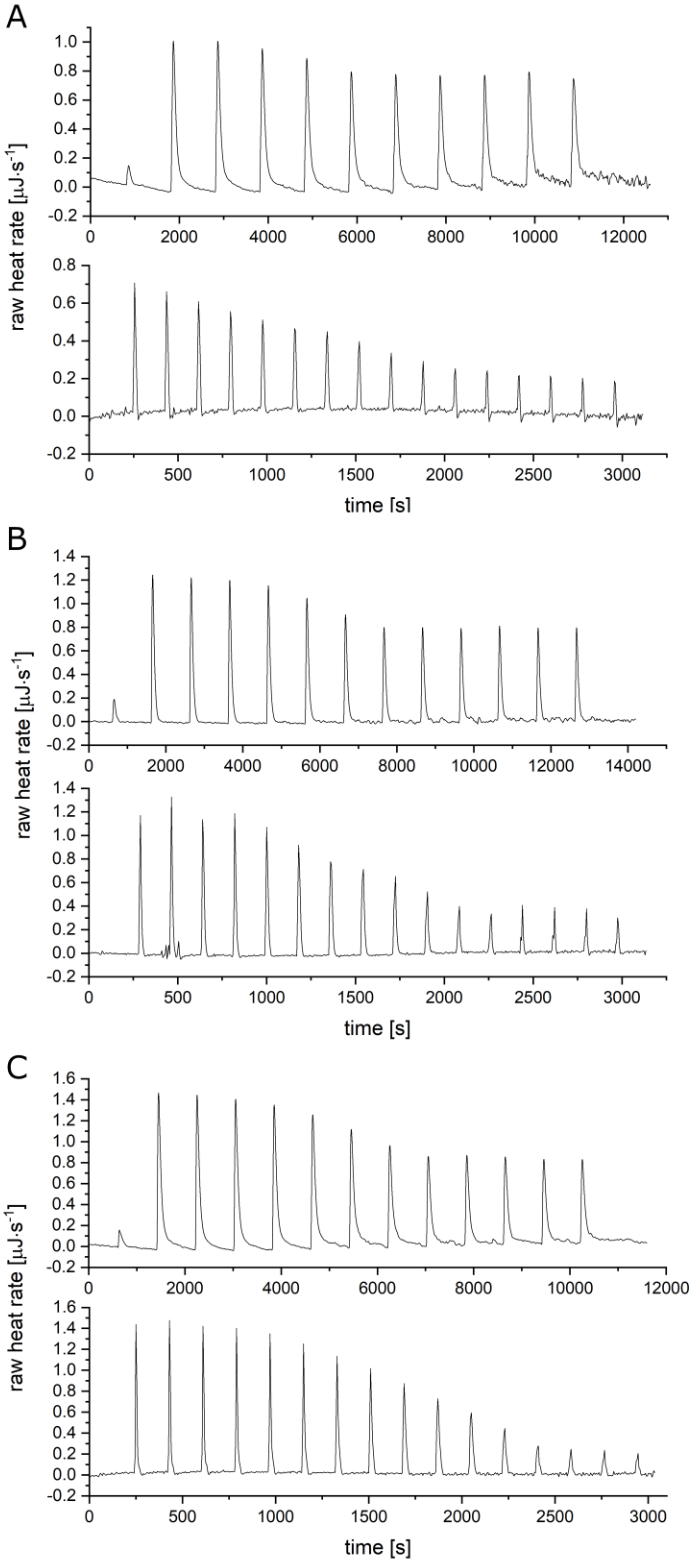

Figure S5. Raw thermograms for binding of TBBt (A), 4,5,6-Br_3Bt (B) and 5,6-Br_2Bt (C) by hCK2α. The data was obtained at 25 °C with NanoITC (TA instruments) and MicroCal iTC200 (Malvern) calorimeters (top and bottom panels, respectively).

Figure S6.

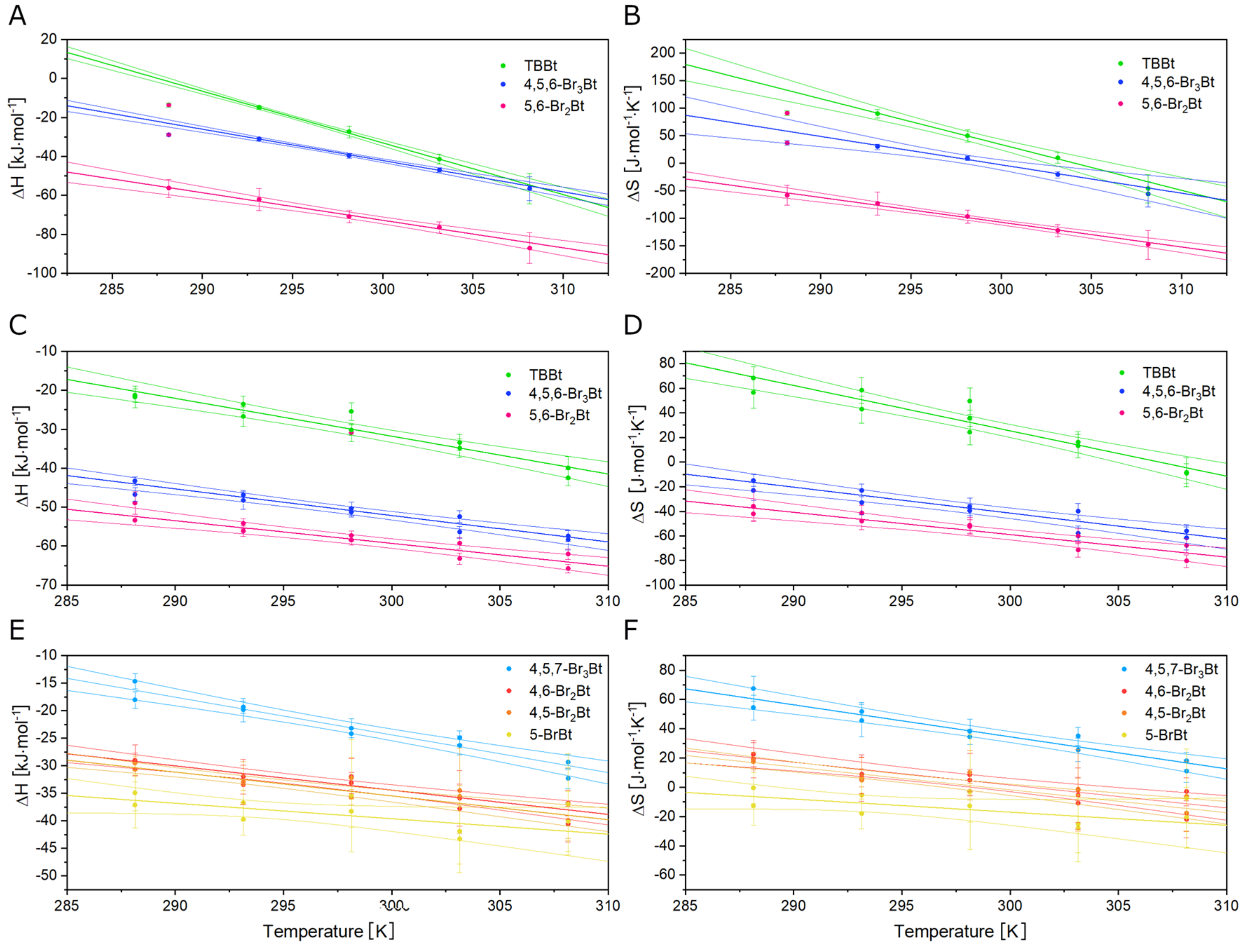

Figure S6. Apparent thermodynamic parameters Δ*H* [kJ·mol^{-1}] (left panel) and Δ*S* [J·mol^{-1}·K^{-1}] (right panel) for binding of bromobenzotriazoles to hCK2α at different temperatures. For the strongest ligands (5,6-Br_2Bt, 4,5,6-Br_3Bt and TBBt), the data were collected with the NanoITC (A,B) and iTC_{200} (C,D) calorimeters. For the remaining ligands (5-BrBt, 4,6-Br_2Bt, 4,5-Br_2Bt and 4,5,7-Br_3Bt), only the iTC_{200} calorimeter could be used (E,F). Thick lines denote the best fit according to linear regression, and thin lines indicate the 95% confidence band for the fitted line. The $\Delta C_{p,\mathrm{bind}}$ values are the derivative of Δ*H* with respect to temperature, i.e. $\Delta C_{p,\mathrm{bind}}=(\partial\Delta H/\partial T)_{\mathrm{p=const}}$.

Figure S7.

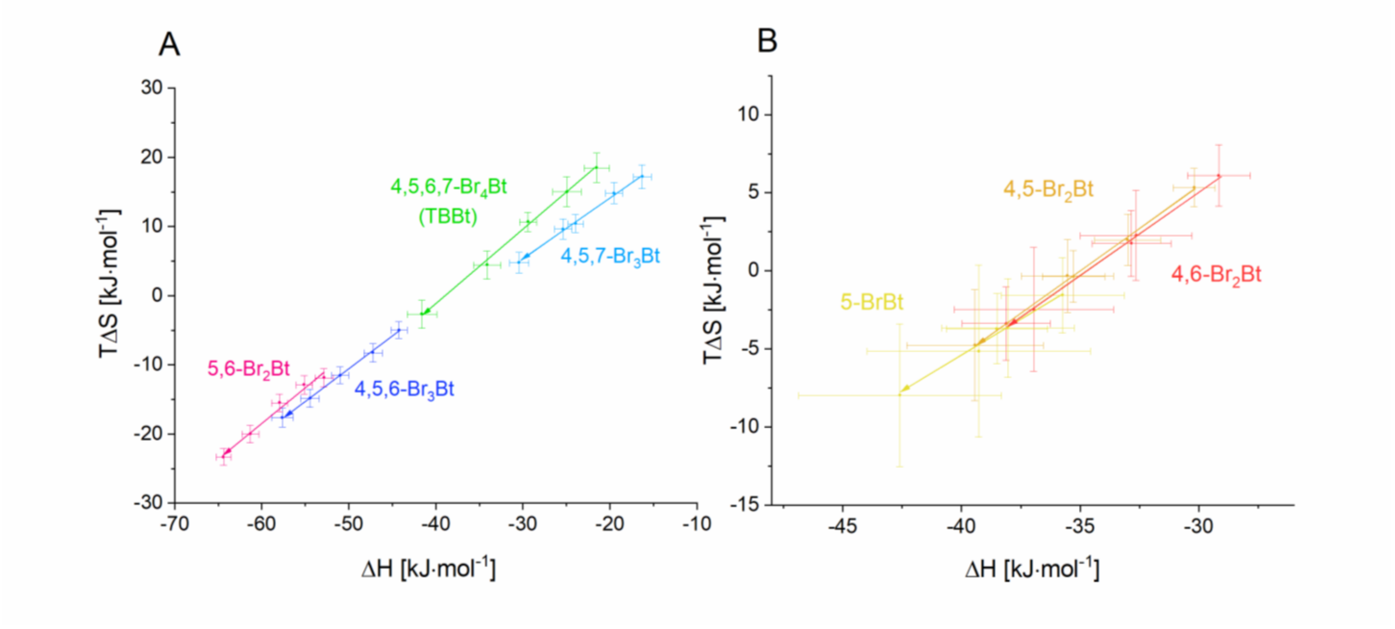

Figure S7. Entropy-enthalpy compensation for hCK2α-bromobenzotriazole interaction. (A,B) Entropy-enthalpy compensation with respect to temperature. Enthalpic and entropic contributions to ligand binding were measured at different temperatures (15, 20, 25, 30, and 35 °C). The arrows mark the increase in the temperature). Entropic Δ*S* and enthalpic –Δ*H* contributions decrease and increase strongly with temperature, respectively. The two effects partially compensate for the free energy of binding Δ*G* = Δ*H* - *T*Δ*S*.

Figure S8.

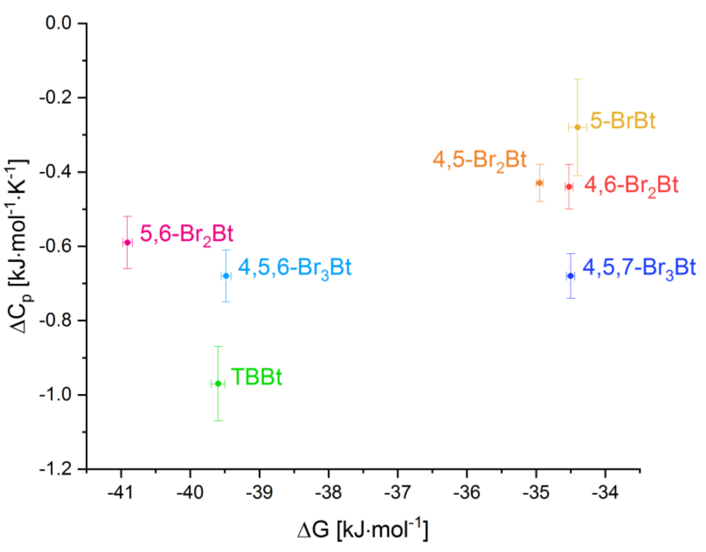

Figure S8. A comparison between the binding affinities of halogenated benzotriazoles (Δ*G*) determined at 25°C and the heat capacity changes associated with ligand binding ($\Delta C_{p,\mathrm{bind}}$).

Figure S9.

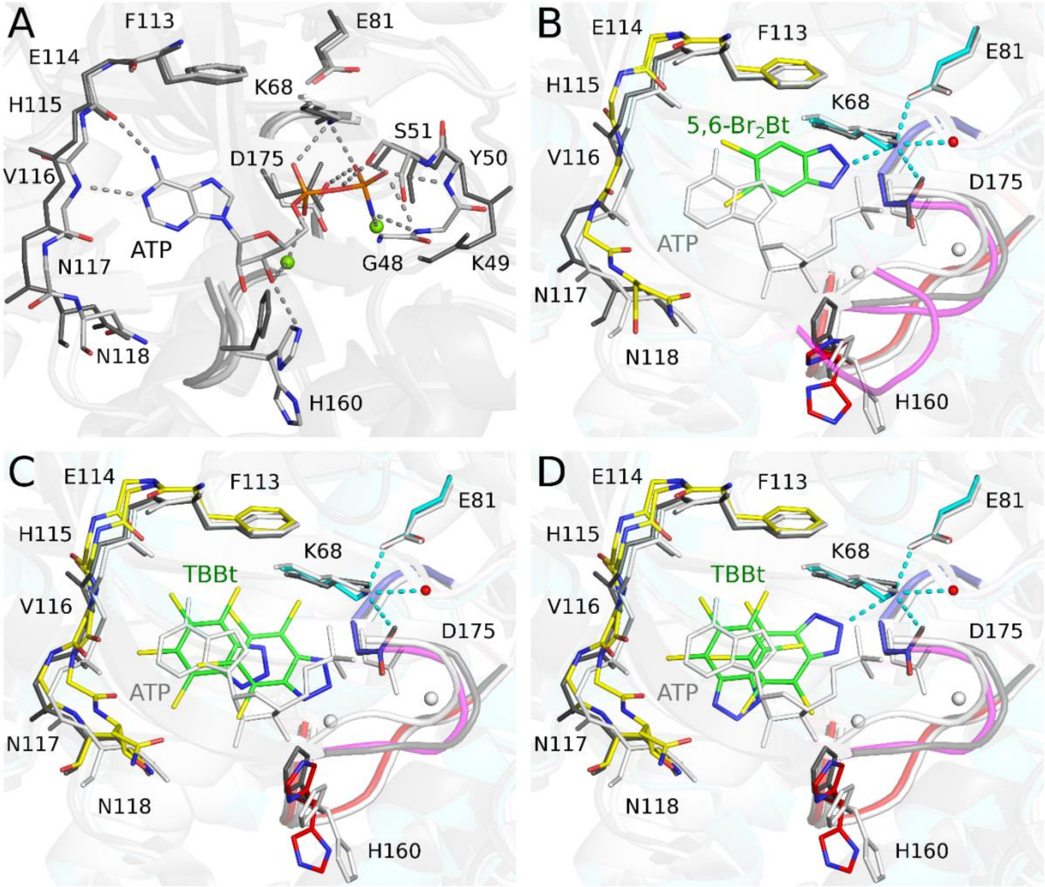

Figure S9. Comparison of the hCK2α in complex with the ATP analogue with (A) the enzyme in the ligand free form, and the two structures overlaid with (B) 5,6-Br$_2$Bt as a representative of the "canonical" binding mode and (C,D) TBBt as an example of the "non-canonical" binding to hCK2α. The complex of hCK2α with the ATP analogue (in white, PDB code: 3NSZ[2]) was superposed on **(A)** the enzyme in the absence of the co-substrate (in gray, PDB code: 3AT2[1]) and then both structures were overlaid on the complexes with **(B)** 5,6-Br$_2$Bt and **(C,D)** TBBt. The major **(C)** and minor **(D)** conformers of TBBt are shown separately for clarity.

Figure S10.

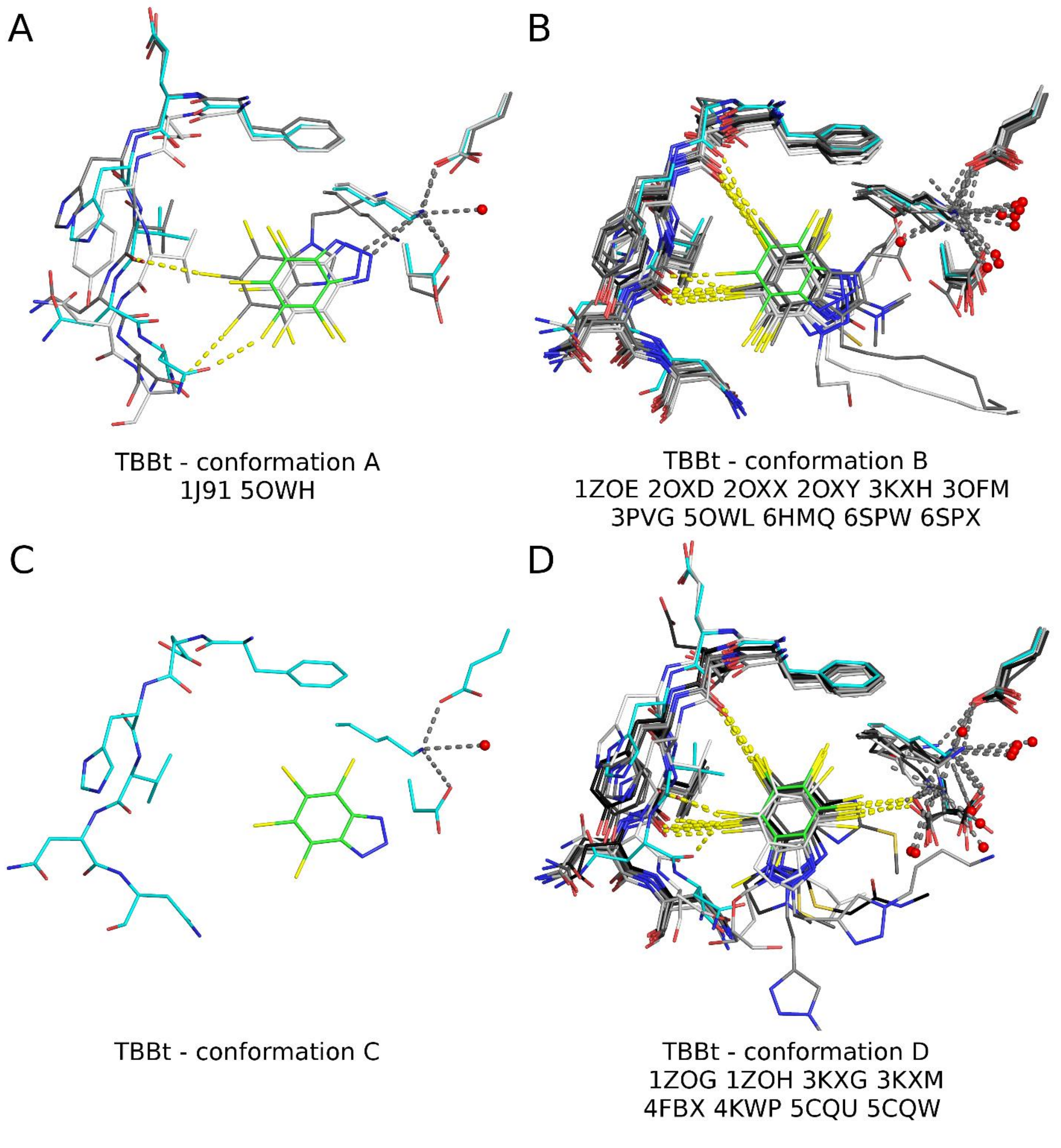

Figure S10. Comparison of the (A-D) TBBt poses observed in the structure of the hCK2α-TBBt complex presented in this work (TBBt in green and yellow, protein in cyan) with the conformations published before (in shades of grey). PDB codes of the other complexes of tetrabromobenzotriazole derivatives and analogues are stated below the four panels. The binding mode of one compound, tetrabromo-1H-benzotriazol-1-yl)propan-1-ol (PDB code: 3RPS) could not be clearly grouped with any of the four conformations but falls in between of the binding modes B and D.

Figure S11.

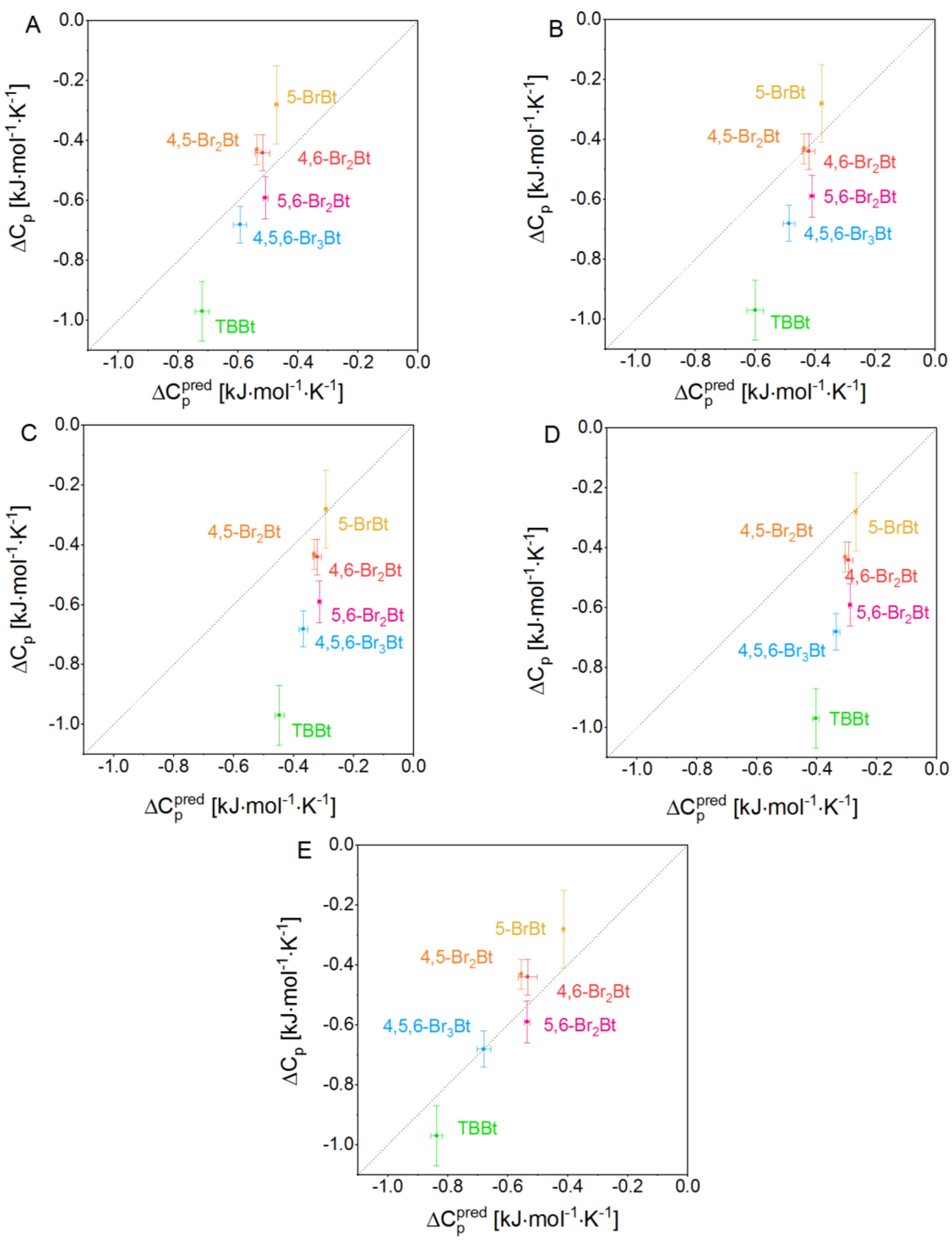

Figure S11. Structure-based predictions of ΔC_p calculated using the change of ligand solvent accessible surface upon complex formation. Δ*ASA* values were extracted with the Yasara Structure package and averaged for all alternative ligand poses based on the crystallographic occupancies. The obtained values were converted to ΔC_p according to the parameterization proposed by **(A)** Makhatadze and Privalov,[7] **(B)** Murphy and Freire,[8] **(C)** Spolar et al.,[9] **(D)** Myers et al.[10] and **(E)** Spolar parameterization extended for the contribution of bromine atom by 3.47 kJ·mol^{-1}·K^{-1}·Å^{-2}.

Figure S12.

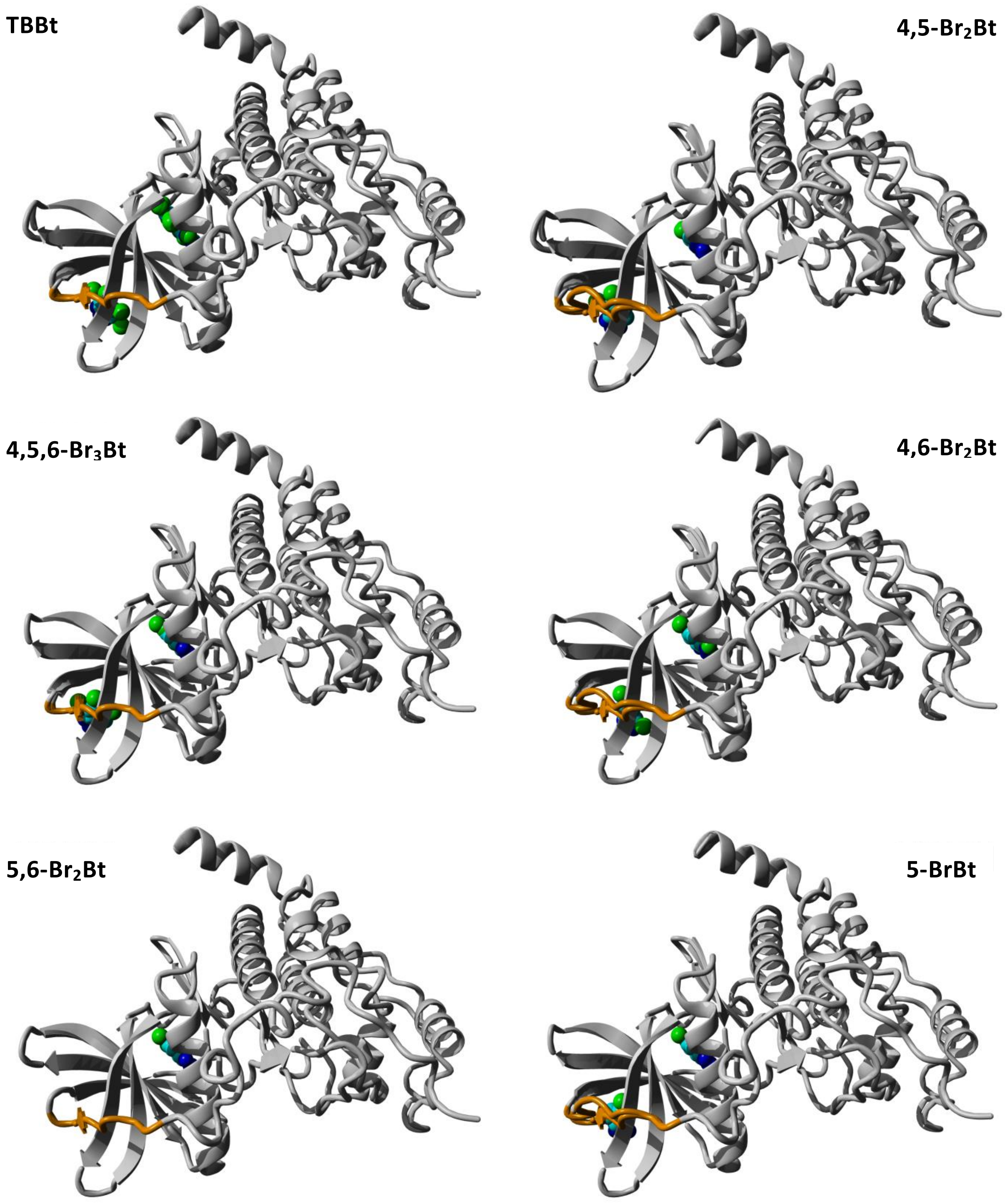

Figure S12. Flexibility of [30]VVEWGNQD[37] hCK2α fragment in the complexes with brominated benzotriazoles. For three strongly binding ligands the fragment is rigid (left panels, orange), while in three weak complexes (right) it displays multiple conformations.

Supplementary Tables

Table S1. Comparison of NanoITC and iTC200 microcalorimeters.

NanoITC		iTC200	
**Specifications**			
Active Cell Volume:	1.0 mL	Active Cell Volume:	200 µL
Injection Syringe Volume	250 µL	Injection Syringe Volume	40 µL
Minimum Injection Volume	0.26 µL	Minimum Injection Volume	0.1 µL
Minimum Detectable Heat:	0.1 µJ	Minimum Detectable Heat:	0.04 µcal (~0.17 µJ)
Maximum Measureable Heat	5 mJ	Maximum Measureable Heat	10 µcal (~42 µJ)
Response Time:	18 sec	Response Time:	10 sec
**Baseline determination method:**			
Linear baseline determination between two points at the beginning and end of integration region		Linear baseline approximation for 24 points, which includes the central peak and each neighboring peak	
**Standard reaction:**			
18-crown-6/$BaCl_2$, 25 °C literature values: K_b= (5.90 ± 0.20)·10^3 M; Δ*H*= – **31.42 ± 0.20 kJ·mol^{-1}** [11]			
K = (5.80 ± 0.43)·10^3 M; Δ*H*= – 27.89 ± 0.63 kJ·mol^{-1}		*K* = (5.38 ± 0.66)·10^3 M; Δ*H*= – 32.5 ± 1.6 kJ·mol^{-1}	

Table S2. Thermodynamic parameters of the bromobenzotriazole compounds with the highest affinity to hCK2α determined with the aid of isothermal titration calorimetry (ITC). The data were collected in two or more independent experiments that were separately evaluated.

NanoITC					iTC200				
T [°C]	ΔG [kJ·mol^{-1}]	Kd [nM]	ΔH [kJ·mol^{-1}]	ΔS [J·mol^{-1}·K^{-1}]	T [°C]	ΔG [kJ·mol^{-1}]	Kd [nM]	ΔH [kJ·mol^{-1}]	ΔS [J·mol^{-1}·K^{-1}]
**4,5,6,7-Br_4Bt (TBBt)**									
15	-40.7 ± 2.9	75 ± 38	-13.7 ± 1.0	91 ± 4	15	-42.4 ± 1.5<br>-39.6 ± 1.5	37 ± 28<br>120 ± 91	-21.4 ± 1.7<br>-21.8 ± 2.8	71 ± 9<br>59 ± 12
20	-41.2 ± 2.1	46 ± 33	-15.0 ± 0.9	90 ± 8	20	-39.9 ± 1.2<br>-41.4 ± 1.4	103 ± 57<br>56 ± 36	-26.7 ± 2.5<br>-23.7 ± 2.1	44 ± 11<br>59 ± 10
25	-42.3 ± 1.4	39 ± 21	-27.5 ± 3.1	50 ± 11	25	-40.2 ± 1.3<br>-38.1 ± 0.9<br>-40.8 ± 0.9	91 ± 49<br>209 ± 83<br>70 ± 29	-25.5 ± 2.2<br>-31.0 ± 2.2<br>-30.2 ± 1.3	49 ± 11<br>24 ± 10<br>36 ± 6
30	-43.8 ± 1.9	28 ± 20	-41.4 ± 2.6	10 ± 10	30	-38.2 ± 0.8<br>-37.7 ± 0.8	203 ± 73<br>248 ± 87	-34.9 ± 2.4<br>-33.5 ± 2.1	11 ± 10<br>14 ± 9
35	-42.4 ± 1.3	65 ± 32	-56.6 ± 7.8	-46 ± 26	35	-38.3 ± 0.6<br>-36.1 ± 0.8	195 ± 55<br>470 ± 150	-42.5 ± 2.1<br>-39.9 ± 2.9	-14 ± 8<br>-30 ± 7
**4,5,6-Br_3Bt**									
15	-39.7 ± 0.5	65 ± 13	-29.0 ± 0.6	37 ± 4	15	-40.3 ± 0.5<br>-41.6 ± 0.8	89 ± 19<br>51 ± 17	-43.3 ± 1.1<br>-46.7 ± 1.8	-10 ± 5<br>-17 ± 8
20	-39.8 ± 0.6	81 ± 19	-31.1 ± 0.9	30 ± 5	20	-39.6 ± 0.5<br>-39.3 ± 0.7	114 ± 22<br>130 ± 38	-46.9 ± 1.2<br>-48.3 ± 2.3	-24 ± 5<br>-30 ± 10
25	-42.1 ± 1.0	42 ± 17	-39.6 ± 1.3	9 ± 4	25	-39.4 ± 0.5<br>-39.6 ± 0.6	123 ± 23<br>116 ± 27	-51.3 ± 1.2<br>-50.4 ± 1.7	-40 ± 5<br>-36 ± 7
30	-40.7 ± 0.5	96 ± 17	-47.0 ± 1.2	-21 ± 6	30	-38.4 ± 0.4<br>-39.7 ± 0.5	189 ± 31<br>109 ± 24	-56.5 ± 1.5<br>-52.4 ± 1.5	-61 ± 6<br>-42 ± 6
35	-39.3 ± 1.1	217 ± 94	-56.5 ± 6.1	-56 ± 23	35	-38.8 ± 0.4<br>-38.1 ± 0.6	160 ± 26<br>215 ± 48	-57.4 ± 1.4<br>-58.4 ± 2.5	-63 ± 5<br>-68 ± 10
**5,6-Br_2Bt**									
15	-39.9 ± 2.0	59 ± 49	-56.4 ± 4.7	-58 ± 18	15	-39.9 ± 0.6<br>-42.7 ± 0.6	101 ± 22<br>33 ± 7	-48.9 ± 2.9<br>-53.4 ± 1.1	-30 ± 12<br>-36 ± 5
20	-40.6 ± 2.2	60 ± 54	-62.1 ± 5.6	-73 ± 21	20	-42.8 ± 0.5<br>-42.7 ± 0.5	32 ± 6<br>33 ± 6	-54.2 ± 1.3<br>-56.2 ± 1.4	-38 ± 6<br>-45 ± 7
25	-41.9 ± 1.4	46 ± 26	-70.9 ± 3.2	-97 ± 12	25	-41.7 ± 0.5<br>-40.4 ± 0.4	50 ± 8<br>27 ± 5	-57.4 ± 1.3<br>-58.5 ± 1.3	-53 ± 6<br>-51 ± 6
30	-39.5 ± 1.9	150 ± 120	-76.5 ± 2.9	-122 ± 11	30	-40.9 ± 0.4<br>-39.8 ± 0.4	84 ± 13<br>69 ± 12	-59.3 ± 1.4<br>-63.3 ± 1.4	-63 ± 6<br>-75 ± 6
35	-41.5 ± 1.3	93 ± 49	-87.0 ± 7.8	-148 ± 26	35	-39.6 ± 0.4<br>-40.4 ± 0.4	108 ± 15<br>115 ± 14	-62.1 ± 1.4<br>-65.8 ± 1.1	-75 ± 6<br>-88 ± 5
**4,5,7-Br_3Bt**									
					15	-35.2 ± 1.1	670 ± 260	-14.7 ± 1.4	69 ± 8

						-34.9 ± 0.9	760 ± 240	-18.1 ± 1.5	56 ± 8
					20	-33.8 ± 1.0<br>-35.1 ± 0.7	1200 ± 440<br>710 ± 180	-19.9 ± 2.1<br>-19.4 ± 1.1	47 ± 11<br>53 ± 6
					25	-34.5 ± 0.5<br>-34.5 ± 0.8	900 ± 160<br>910 ± 250	-24.2 ± 1.0<br>-23.2 ± 1.7	34 ± 5<br>38 ± 9
					30	-33.5 ± 0.6<br>-34.9 ± 0.6	1360 ± 300<br>760 ± 160	-26.3 ± 1.7<br>-24.9 ± 1.2	24 ± 8<br>34 ± 6
					35	-33.7 ± 0.4<br>-34.6 ± 0.5	1230 ± 220<br>870 ± 180	-29.3 ± 1.4<br>-32.3 ± 1.8	15 ± 6<br>8 ± 6
**4,6-Br_2Bt**									
					15	-36.9 ± 0.7<br>-35.8 ± 0.9	343 ± 84<br>540 ± 200	-29.2 ± 1.5<br>-29.0 ± 2.8	26 ± 8<br>23 ± 12
					20	-35.7 ± 0.9<br>-35.2 ± 0.9	550 ± 210<br>680 ± 250	-33.5 ± 3.6<br>-32.0 ± 3.1	7 ± 15<br>11 ± 13
					25	-34.5 ± 0.5<br>-34.6 ± 0.9	900 ± 200<br>880 ± 320	-33.1 ± 1.9<br>-32.0 ± 3.4	5 ± 8<br>9 ± 15
					30	-33.7 ± 0.9<br>-33.9 ± 0.9	1250 ± 490<br>1140 ± 420	-35.9 ± 5.0<br>-37.8 ± 4.5	-7 ± 19<br>-13 ± 18
					35	-34.7 ± 0.6<br>-32.1 ± 2.6*<br>-32.8 ± 0.6	840 ± 200<br>2400 ± 2500*<br>1770 ± 400	-36.8 ± 2.3<br>-40 ± 18*<br>-40.6 ± 3.2	-7 ± 10<br>-26 ± 71*<br>-26 ± 13
**4,5-Br_2Bt**									
					15	-37.3 ± 0.5<br>-36.1 ± 0.5	260 ± 53<br>470 ± 100	-30.7 ± 1.2<br>-29.6 ± 1.3	21 ± 6<br>22 ± 6
					20	-35.5 ± 0.5<br>-35.8 ± 0.8	610 ± 120<br>540 ± 180	-33.0 ± 1.5<br>-33.0 ± 3.7	8 ± 6<br>7 ± 15
					25	-34.9 ± 0.4<br>-35.2 ± 0.9	770 ± 130<br>670 ± 260	-35.7 ± 1.4<br>-32.2 ± 3.8	-3 ± 6<br>10 ± 15
					30	-34.6 ± 0.5<br>-33.4 ± 1.3	880 ± 180<br>1430 ± 810	-35.6 ± 2.0<br>-34.5 ± 7.8	-3 ± 8<br>-4 ± 29
					35	-33.4 ± 0.6<br>-33.9 ± 1.2	1440 ± 340<br>1160 ± 560	-40.0 ± 3.2<br>-37.0 ± 6.6	-22 ± 13<br>-10 ± 25
**5-BrBt**									
					15	-34.6 ± 0.9<br>-36.0 ± 0.8	880 ± 350<br>490 ± 190	-37.1 ± 4.2<br>-34.9 ± 3.3	-9 ± 15<br>4 ± 12
					20	-35.0 ± 0.7<br>-35.7 ± 0.9	730 ± 210<br>550 ± 200	-39.8 ± 2.8<br>-36.7 ± 3.3	-16 ± 12<br>-3 ± 14
					25	-34.4 ± 0.6<br>-34.4 ± 1.5	930 ± 280<br>930 ± 580	-38.3 ± 2.9<br>-35 ± 10	-13 ± 11<br>-3 ± 39
					30	-33.7 ± 1.1<br>-34.3 ± 1.1	1270 ± 550<br>990 ± 440	-41.9 ± 6.0<br>-43.3 ± 6.1	-28 ± 20<br>-30 ± 24
					35	-33.5 ± 1.6<br>-32.7 ± 0.9	1330 ± 860<br>1890 ± 650	-37.0 ± 9.1<br>-40.1 ± 5.5	-12 ± 36<br>-25 ± 21

***** This data was excluded from further analysis because of excessive errors.

Table S3. ITC-derived heat capacity change associated with ligand binding ($\Delta C_{p,\mathrm{bind}}$), and the reduction of solvent accessible surface upon ligand binding (ΔASA) estimated from the structural data of their complexes. ΔASA data were converted into $\Delta C_{p,\mathrm{bind}}$ using four alternative weights for polar and nonpolar surfaces, one of which was additionally extended for the contribution of bromine.

Ligand	ΔASA [Å^2]			$\Delta C_{p,\mathrm{bind}}$ [kJ·mol^{-1}·K^{-1}]					
	Polar	Non-polar	Br	Makhatadze & Privalov[7]	Murphy & Freire[8]	Spolar et al.[9]	Myers et al.[10]	Spolar et al.[9]+Br	ITC
**4,5,6,7-Br$_4$Bt**	101±23	195±3	182±4	-0.72±0.02	-0.60±0.03	-0.44±0.02	-0.40±0.01	-0.84±0.02	-0.97 ± 0.10
**4,5,7-Br$_3$Bt**				-	-	-	-	-	-0.68 ± 0.07
**4,5,6-Br$_3$Bt**	106±1	174±9	146±6	-0.59±0.02	-0.49±0.02	-0.37±0.01	-0.34±0.01	-0.68±0.02	-0.68 ± 0.06
**5,6-Br$_2$Bt**	111±1	179±3	103±2	-0.51±0.01	-0.41±0.01	-0.31±0.01	-0.29±0.00	-0.54±0.01	-0.59 ± 0.07
**4,5-Br$_2$Bt**	109±3	191±0	104±0	-0.54±0.00	-0.44±0.00	-0.33±0.00	-0.30±0.00	-0.55±0.00	-0.43 ± 0.05
**4,6-Br$_2$Bt**	105±3	186±6	99±9	-0.52±0.02	-0.42±0.02	-0.32±0.01	-0.29±0.01	-0.53±0.03	-0.44 ± 0.06
**4,7-Br$_2$Bt**	92±4	219±1	107±2	-0.62±0.01	-0.52±0.01	-0.38±0.00	-0.35±0.00	-0.61±0.01	-
**5-BrBt**	111±1	208±3	57±0	-0.47±0.01	-0.38±0.01	-0.29±0.00	-0.27±0.00	-0.41±0.00	-0.28 ± 0.13
**4-BrBt**	103±3	218±9	47±7	-0.48±0.02	-0.39±0.02	-0.30±0.02	-0.27±0.01	-0.40±0.03	-

Table S4. Entropic contributions to the binding of bromobenzotriazoles by hCK2α. This is the temperature, at which the entropy change upon ligand binding is zero; ΔS_{rt} is the unfavorable entropic term reflecting the reduction of ligand rotational and translational degrees of freedom upon binding; $\Delta S_{HE}(T_s)$ is the contribution to the entropy change upon ligand binding from the hydrophobic effect; ΔS_{other} is the entropy change assigned to all other processes accompanying ligand binding.

Ligand	ΔS_{rt}[a] [J·mol^{-1}·K^{-1}]	T_s[b] [K]	$\Delta S_{HE}(T_s)$[c] [J·mol^{-1}·K^{-1}]	ΔS_{other}[d] [J·mol^{-1}·K^{-1}]
**4,5,6,7-Br_4Bt**	-310	307 ± 30	300 ± 15	10 ± 15
**4,5,6-Br_3Bt**	-301	280 ± 36	295 ± 30	6 ± 30
**5,6-Br_2Bt**	-291	268 ± 42	291 ± 34	0 ± 34
**4,5,7-Br_3Bt**	-301	316 ± 33	184 ± 16	118 ± 16
**4,6-Br_2Bt**	-291	301 ± 47	148 ± 20	143 ± 20
**4,5-Br_2Bt**	-291	299 ± 37	148 ± 17	143 ± 17
**5-BrBt**	-277	281 ± 152	120 ± 56	157 ± 56

[a] $\Delta S_{rt} \approx 57$ J·mol^{-1}·K^{-1} + $5 \cdot R \cdot \ln(m \cdot Da^{-1})$[12];

[b] Estimated from the linear fit of $\Delta S(T)$ (Fig. S6 DF);

[c] $\Delta S_{HE}(T_s) \approx 1.35 \cdot \Delta C_{p,bind} \cdot \ln(T_s/386)$ [13];

[d] $\Delta S_{rt} + \Delta S_{HE}(T_S) + \Delta S_{other} = 0$[12].

References

1. Kinoshita, T.; Sekiguchi, Y.; Fukada, H.; Nakaniwa, T.; Tada, T.; Nakamura, S.; Kitaura, K.; Ohno, H.; Suzuki, Y.; Hirasawa, A., et al., A Detailed Thermodynamic Profile of Cyclopentyl and Isopropyl Derivatives Binding to CK2 Kinase. *Mol. Cell. Biochem.* **2011**, *356*, 97-105.
2. Ferguson, A. D.; Sheth, P. R.; Basso, A. D.; Paliwal, S.; Gray, K.; Fischmann, T. O.; Le, H. V., Structural Basis of Cx-4945 Binding to Human Protein Kinase CK2. *FEBS Lett.* **2011**, *585*, 104-110.
3. Raaf, J.; Brunstein, E.; Issinger, O. G.; Niefind, K., The CK2 Alpha/CK2 Beta Interface of Human Protein Kinase CK2 Harbors a Binding Pocket for Small Molecules. *Chem. Biol.* **2008**, *15*, 111-117.
4. Niefind, K.; Guerra, B.; Ermakowa, I.; Issinger, O. G., Crystal Structure of Human Protein Kinase CK2: Insights into Basic Properties of the CK2 Holoenzyme. *EMBO J.* **2001**, *20*, 5320-5331.
5. Cheng, Y.; Prusoff, W. H., Relationship between Inhibition Constant (K_i) and Concentration of Inhibitor Which Causes 50 Per Cent Inhibition (I_{50}) of an Enzymatic-Reaction. *Biochem. Pharmacol.* **1973**, *22*, 3099-3108.
6. Winiewska, M.; Makowska, M.; Maj, P.; Wielechowska, M.; Bretner, M.; Poznanski, J.; Shugar, D., Thermodynamic Parameters for Binding of Some Halogenated Inhibitors of Human Protein Kinase CK2. *Biochem. Biophys. Res. Commun.* **2015**, *456*, 282-287.
7. Makhatadze, G. I.; Privalov, P. L., Energetics of Protein Structure. In *Advances in Protein Chemistry, Vol 47*, Anfinsen, C. B.; Edsall, J. T.; Richards, F. M.; Eisenberg, D. S., Eds. 1995; Vol. 47, pp 307-425.
8. Murphy, K. P.; Freire, E., Thermodynamics of Structural Stability and Cooperative Folding Behavior in Proteins. *Adv. Protein Chem.* **1992**, *43*, 313-361.
9. Spolar, R. S.; Livingstone, J. R.; Record, M. T., Use of Liquid-Hydrocarbon and Amide Transfer Data to Estimate Contributions to Thermodynamic Functions of Protein Folding from the Removal of Nonpolar and Polar Surface from Water. *Biochemistry* **1992**, *31*, 3947-3955.
10. Myers, J. K.; Pace, C. N.; Scholtz, J. M., Denaturant M-Values and Heat-Capacity Changes - Relation to Changes in Accessible Surface-Areas of Protein Unfolding. *Protein Sci.* **1995**, *4*, 2138-2148.
11. Wadso, I.; Goldberg, R. N., Standards in Isothermal Microcalorimetry (IUPAC Technical Report). *Pure Appl. Chem.* **2001**, *73*, 1625-1639.
12. Murray, C. W.; Verdonk, M. L., The Consequences of Translational and Rotational Entropy Lost by Small Molecules on Binding to Proteins. *J. Comput. Aid. Mol. Des.* **2002**, *16*, 741-753.
13. Spolar, R. S.; Record, M. T., Coupling of Local Folding to Site-Specific Binding of Proteins to DNA. *Science* **1994**, *263*, 777-784.
